# Antibody-induced internalization and degradation of PLA2R amplifies CD4^+^ T cell activation

**DOI:** 10.7150/thno.123035

**Published:** 2026-01-01

**Authors:** Yuning Liu, Yinyin Li, Hui Zhou, Jieli Yu, Pei Deng, Weiwei Xu, Bingqian Pan, Lei Zhang, Hong Zhou, Jing Zhang, Xiaohan Liu, Min Shi, Xianchi Dong, Bei Tong, Enguang Bi, Nannan Guo, Yu Hu

**Affiliations:** 1 Division of Nephrology, Nanfang Hospital, Southern Medical University; State Key Laboratory of Multi-organ Injury Prevention and Treatment; National Clinical Research Center for Kidney Disease; Guangdong Provincial Institute of Nephrology; Guangdong Provincial Key Laboratory of Renal Failure Research, Guangzhou, Guangdong 510515, China.; 2 Department of Laboratory Medicine, Nanfang Hospital, Southern Medical University, Guangzhou, Guangdong 510515, China.; 3 Department of Oncology, Nanfang Hospital, Southern Medical University, Guangzhou, Guangdong 510515, China.; 4 School of Life Science, Nanjing University, Nanjing, Jiangsu 210023, China.; 5 Engineering Research Center of Protein and Peptide Medicine, Ministry of Education, Nanjing, Jiangsu 210023, China.; 6 Jiangsu Key Laboratory for Conservation and Utilization of Plant Resources, Institute of Botany, Jiangsu Province and Chinese Academy of Sciences (Nanjing Botanical Garden Memorial Sun Yat-Sen), Nanjing 210014, China.; 7 Department of Biochemistry and Molecular Biology, School of Basic Medical Sciences, Guangdong Provincial Key Laboratory of Single Cell Technology and Application, Southern Medical University, Guangzhou, Guangdong 510515, China.; 8 Key Laboratory of Mental Health of the Ministry of Education, Guangdong-Hong Kong-Macao Greater Bay Area Center for Brain Science and Brain-Inspired Intelligence, Guangdong-Hong Kong Joint Laboratory for Psychiatric Disorders, Guangdong Province Key Laboratory of Psychiatric Disorders, Guangdong Basic Research Center of Excellence for Integrated Traditional and Western Medicine for Qingzhi Diseases, Department of Neurobiology, School of Basic Medical Sciences, Southern Medical University, Guangzhou, Guangdong 510515, China.

**Keywords:** primary membranous nephropathy, PLA2R, clathrin-mediated endocytosis, antigen presentation, CD4⁺ T cells

## Abstract

**Rationale:** Phospholipase A2 receptor (PLA2R) is the predominant autoantigen in primary membranous nephropathy (PMN), accounting for approximately 70% of clinical cases. However, the mechanisms by which PLA2R initiates and sustains autoimmunity in PMN remain unclear. PLA2R belongs to the mannose receptor (MR) family, members of which have been shown to undergo endocytosis and lysosomal degradation for MHCII-mediated antigen presentation. This study investigates whether antibody binding promotes PLA2R internalization and lysosomal processing to enhance MHCII-mediated antigen presentation and CD4⁺ T cell activation, thereby contributing to the perpetuation of autoimmunity in PMN.

**Methods:** Multiple PLA2R-overexpressing cell lines were generated by lentiviral-mediated overexpression of PLA2R. Imaging and western blot were employed to assess the effects of anti-PLA2R antibodies, derived from PMN patients or produced in-house, on PLA2R internalization and degradation. To define the specific endocytic pathway involved, we used pharmacological inhibitors of endocytosis as well as PLA2R constructs lacking the endocytic domain. Finally, T cell activation was evaluated using OT-II CD4⁺ T cells co-cultured with PLA2R-ovalbumin (OVA)-expressing mouse dendritic cells treated with anti-PLA2R antibodies.

**Results:** Binding of anti-PLA2R antibodies triggers clathrin-mediated endocytosis and lysosomal trafficking of PLA2R. Antibody-induced PLA2R degradation was effectively prevented by specific endocytosis inhibitors or by deletion of the PLA2R endocytic domain. Furthermore, PLA2R-OVA-expressing mouse dendritic cells exposed to PLA2R antibodies enhanced the activation of OVA-specific CD4⁺ T cells both *in vitro* and *in vivo*.

**Conclusions:** This study demonstrates that anti-PLA2R antibody induces internalization and lysosomal degradation of PLA2R, a process that may enhance MHC class II-mediated antigen presentation and promote the expansion of antigen-specific CD4⁺ T cells. This mechanism could establish a self-reinforcing feedback loop that perpetuates autoimmune responses in PMN.

## Introduction

Primary membranous nephropathy (PMN) is a leading cause of nephrotic syndrome in adults, characterized by autoantibody-mediated damage to the kidney's filtration system 1-4. Unlike secondary membranous nephropathy (MN), which is associated with underlying conditions such as infections, cancer, systemic autoimmune diseases, and drug reactions, PMN arises primarily from the immune system's targeting of specific antigens on podocytes. The autoantibodies, particularly those against phospholipase A2 receptor (PLA2R) and thrombospondin type-1 domain-containing 7A (THSD7A) 5, 6, form immune complexes in the subepithelial space of the glomerular basement membrane (GBM). The accumulation of these complexes leads to GBM thickening, complement activation, and progressive podocyte injury, ultimately resulting in proteinuria - a hallmark of nephrotic syndrome.

The development of antibodies targeting podocyte antigens, such as PLA2R, is a critical initiating factor in the pathogenesis of PMN. High levels of PLA2R antibodies have been shown to correlate with increased proteinuria at baseline and a reduced likelihood of spontaneous remission 7, 8. In clinical practice, therapeutic strategies aimed at depleting PLA2R antibodies, such as rituximab treatment, have been effective in inducing remission of nephrotic syndrome in PMN patients 9. The disappearance of PLA2R antibodies often precedes clinical remission, underscoring the key pathogenic role of these antibodies in the disease 9, 10. Conversely, patients who fail to reduce PLA2R antibody levels generally do not experience a corresponding reduction in proteinuria 9.

Despite the identification of more than a dozen antigens associated with membranous nephropathy (MN) 1, 5, 6, 11, PLA2R remains the most prevalent, as approximately 70% of PMN patients test positive for PLA2R antibodies 12. However, the mechanisms driving the generation of PLA2R-specific autoimmunity are still not fully understood. Emerging evidence, particularly from genetic association studies, suggests that CD4^+^ T cells play a key role in the pathogenesis of PMN 13-18. Genetic predisposition to PMN is strongly associated with specific loci, including the PLA2R gene and the human leukocyte antigen (HLA) class II region, with the HLA genetic variants having a more significant impact than PLA2R variants 15, 17, 18. Individuals carrying both the PLA2R and HLA-DQA1 risk alleles exhibit an even higher likelihood of developing the disease 13, 14, 17. Several studies have identified single-nucleotide polymorphisms (SNPs) in the HLA-DQA1 and HLA-DRB1 regions that increase the risk of developing PMN. The risk alleles in HLA-DQA1 and HLA-DRB1 are thought to alter the antigen-binding grooves of these MHC class II (MHCII) molecules, potentially enhancing the presentation of PLA2R-derived peptides to CD4^+^ T cells 18. By screening a peptide library, Zhang et al. mapped and identified several T cell epitopes of PLA2R, demonstrating that these epitopes induced significantly greater proliferation of CD4^+^ T cells from PMN patients compared to healthy donors 19. Given the essential role of CD4^+^ T cells in supporting B cell-mediated functions, such as antibody production, affinity maturation, and isotype switching, it is plausible that PLA2R-specific CD4^+^ T cell responses are a prerequisite for the generation of PLA2R autoantibodies in PMN.

PLA2R is a 185 kDa type I transmembrane glycoprotein predominantly expressed on podocytes in the kidney 5, 20. It belongs to the mannose receptor (MR, also called CD206) family, which includes MR, urokinase plasminogen activator receptor-associated protein (uPARAP or Endo180), and DEC-205 21-23. A vital feature of this receptor family is their function as endocytic receptors, facilitating the internalization and delivery of ligands to endocytic or lysosomal compartments 24, 25. For instance, the FnII domain in members like Endo180 and MR mediates the internalization of collagen, followed by its lysosomal degradation. This process contributes to collagen clearance in biological events like bone development, cancer invasion, and fibrosis 26-28. Notably, collagen internalization is specific to Endo180 and MR but does not occur in PLA2R or DEC-205 26, highlighting distinct functional roles within the family. MR and DEC-205 are highly expressed in antigen-presenting cells (APCs) like dendritic cells and macrophages, where they facilitate antigen uptake and delivery to lysosomes for processing and presentation. This pathway enables the loading of antigens onto MHCII molecules, promoting efficient CD4^+^ T cell activation. The role of MR in processing antigens and enhancing the potency of APCs has long been established. Meanwhile, DEC-205 undergoes rapid internalization and lysosomal processing upon ligand or antibody binding, leading to significantly enhanced CD4^+^ T cell activation 29-33. PLA2R, while not directly involved in collagen internalization, shares functional similarities with other members of the MR family, such as being internalized via clathrin-coated pits. Through clathrin-mediated endocytosis, PLA2R is internalized upon binding to its ligand, secretory phospholipase A2 (sPLA2) 24, 34. PLA2R has been found on APCs like podocytes, lung macrophages, and neutrophils 35, 36, yet its specific role in immune activation remains unclear. In particular, it is not fully understood how PLA2R antibodies in PMN might induce PLA2R internalization and subsequent antigen presentation to CD4^+^ T cells.

Therefore, we hypothesize that binding of PLA2R by autoantibodies may promote its internalization and subsequent lysosomal degradation, thereby facilitate peptide presentation through MHCII molecules and activate antigen-specific CD4⁺ T cells. To test our hypothesis, we first used imaging and western blot analyses in cellular assays to determine whether PMN patient sera alter the subcellular distribution and expression levels of PLA2R. We then applied PLA2R mutants and pharmacological inhibitors of endocytosis and lysosomal degradation to investigate the molecular mechanisms underlying antibody-induced PLA2R internalization and processing. Finally, to link these processes to T cell immunity, we employed an OT-II CD4⁺ T cell model system to assess whether PLA2R antibody-mediated internalization can enhance antigen processing and promote CD4⁺ T cell activation.

## Results

### PLA2R antibody-positive serum downregulate PLA2R expression

To investigate the effects of PLA2R antibodies (PLA2R-Abs) on PLA2R expression, we aimed to conduct *in vitro* cell-based assays. However, to our knowledge, no cell lines with endogenous PLA2R expression are currently available 37. Therefore, we stably expressed human PLA2R in several cell lines, including MPC5 (mouse podocyte cell line), NIH3T3 (mouse fibroblast cell line), Jurkat (human T cell line) and 293T (human embryonic kidney cell line) (Figure S1A-B). Western blot analysis confirmed successful expression of PLA2R in these cell lines (Figure S1C).

**Figure 1:**
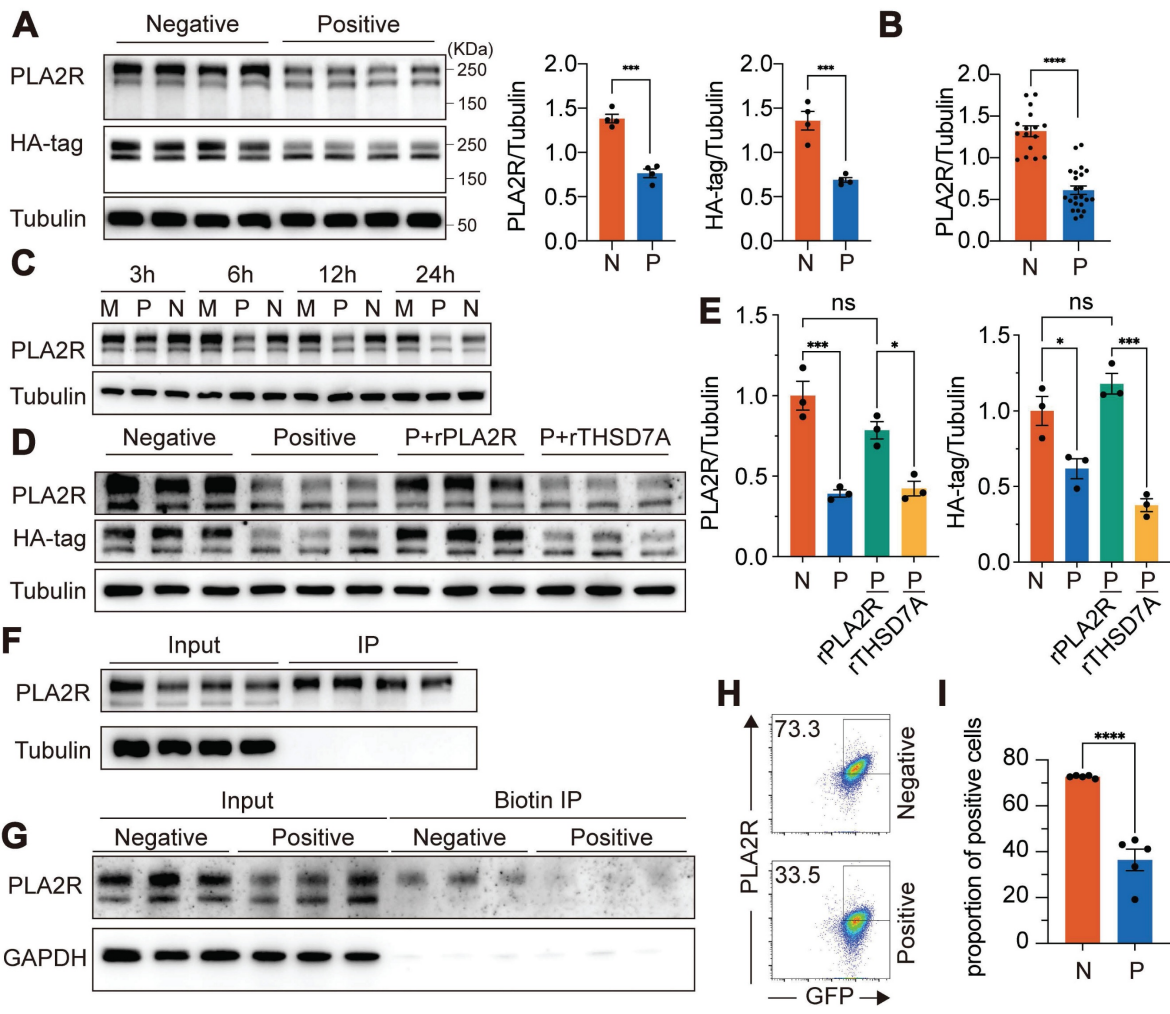
** Antibody recognition mediates PLA2R-Ab^+^ serum-induced downregulation of PLA2R expression.** (**A**) Western blot analysis of PLA2R expression in PLA2R-HA-expressing Jurkat cells (PLA2R-Jurkat) treated for 6 h with PLA2R-Ab^+^ serum samples (Positive, n = 4) or PLA2R-Ab-negative serum from patients with other glomerular diseases (Negative, n = 4). Positive serum was used at a concentration of 5 RU/mL. Negative serum was added equal to the maximum amount of the Positive serum. Representative (left panel) and quantitative data (middle and right panels) are shown. N, Negative; P, Positive. (**B**) Summary of PLA2R expression in Jurkat-PLA2R cells upon treatment with Negative (n = 16) or Positive (n = 23) sera. (**C**) Western blot analysis of PLA2R expression in PLA2R-Jurkat treated for indicated hours with medium only (Mock, M), or Positive or Negative serum. (**D**-**E**) PLA2R-Jurkat cells were incubated with recombinant PLA2R (rPLA2R) or THSD7A for 30 min prior to treatment with Negative (n = 3) or Positive (n = 3) serum. Representative western blot and quantitative data for expression of PLA2R are shown in **D** and **E**, respectively. (**F**) Cell lysate of PLA2R-Jurkat cells was immunoprecipitated with each of four Positive serum. PLA2R levels in cell lysate (Input) or the immunoprecipitates (IP) were analyzed by western blot. (**G**) Surface-biotinylated PLA2R-Jurkat cells were treated with Negative (n = 3) or Positive (n = 3) serum followed by immunoprecipitation with streptavidin-beads. PLA2R levels in cell lysate (Input) or the streptavidin-enriched IP were analyzed by western blot. (**H**-**I**) Flow cytometry detection of PLA2R level on the surface of PLA2R-Jurkat cells treated with Negative (n = 5) or Positive (n = 5) serum. Representative and quantitative data are shown in **H** and **I**, respectively. Data is presented as mean ± SEM from 3-5 individuals. Experiments were repeated 2-3 times with similar results. Statistical significance was assessed using one-way ANOVA followed by Tukey multiple-comparison test (**E**) or 2-tailed, unpaired Student's t test (**A**, **B** and **I**). ns, non-significant; *P < 0.05; ***P < 0.001; ****P < 0.0001.

To evaluate the impact of PLA2R-Abs on PLA2R expression, we added PLA2R-Ab^+^ serum to the PLA2R-Jurkat cell culture. Since previous reports suggest that complement, in the presence of PLA2R-Ab^+^ serum, can degrade podocyte surface proteins such as synaptopodin and NEPH1 37, we inactivated complement by preheating serum at 56 °C for 30 min to eliminate its effect. Using PLA2R-Ab^+^ serum as the primary antibody, we detected PLA2R expression on the plasma membrane of PLA2R-Jurkat cells by flow cytometry, confirming that membrane-localized PLA2R can be recognized by PLA2R-Abs present in the serum of PMN patients (Figure S1D). After incubating PLA2R-Jurkat cells with PLA2R-Ab^+^ serum for various durations, we assessed PLA2R expression in total cell lysates (TCL) via western blot. Our results showed that, compared to Negative serum (from MN patients without detectable PLA2R antibodies), Positive serum (from PMN patients with PLA2R antibodies) significantly reduced PLA2R expression in TCL, as detected by either mouse anti-PLA2R antibody or anti-HA-tag antibody (Figure 1A). We have evaluated total 16 Negative sera and 23 PLA2R-Ab^+^ sera and observed a consistent downregulation of PLA2R upon treatment with PLA2R-Ab^+^ sera (Figure 1B). Notably, reduction in PLA2R expression was first detected between 3 h and 6 h following exposure to the PLA2R-Ab^+^ sera. The downregulation reached its peak at 12 h and persisted through the 24 h mark (Figure 1C). To test whether this downregulation is a universal phenomenon, we repeated the experiment using murine cell lines MPC5 and NIH3T3, and human cell line 293T cells, and observed similar reductions across all cell lines (Figure S1E-G). No significant difference in PLA2R mRNA levels was observed between cells treated with PLA2R-Ab^+^ serum and controls, suggesting that the decrease in PLA2R protein was not due to changes in gene expression (Figure S1H). Moreover, we found a correlation between higher concentrations of PLA2R-Ab^+^ serum and a greater decrease in PLA2R protein levels (Figure S1I). In addition, PLA2R-Ab^+^ serum did not impact the apoptosis of PLA2R-expressing Jurkat and MPC5 cells (Figure S1J-K). To determine whether PLA2R-Ab recognition is required for this effect, we pre-incubated serum samples with recombinant PLA2R protein before exposing them to the cells. Recombinant THSD7A, another PMN autoantigen, was used as a negative control. We found that recombinant PLA2R effectively blocked the downregulation induced by PLA2R-Ab^+^ serum, while THSD7A had no noticeable effect (Figure 1D-E).

Consistent with previous studies 5, 8, we observed two distinct bands representing PLA2R: a high molecular weight form (PLA2R-H) and a low molecular weight form (PLA2R-L) (Figure S1C). Treatment with the deglycosylation enzyme PNGase F collapsed the two bands into one, indicating that the size variation was due to different glycosylation patterns (Figure S1L). Interestingly, PLA2R-Ab^+^ serum specifically reduced the PLA2R-H isoform, with little effect on PLA2R-L (Figure 1A). Since PLA2R-Abs can only access surface-expressed PLA2R, we hypothesized that PLA2R-H is the predominant isoform on the plasma membrane. Therefore, we performed immunoprecipitation (IP) using PLA2R-Ab^+^ serum as capture antibodies. PLAR-Jurkat cells were incubated with the serum on ice to allow binding without triggering endocytosis, ensuring that only surface-expressed PLA2R would interact with the antibodies. After removing excess antibodies, we immunoprecipitated the surface-bound PLA2R-Ab/PLA2R complex with Protein A/G-coupled beads. As shown in Figure 1F, PLA2R-Ab^+^ serum predominantly enriched the PLA2R-H isoform, supporting our hypothesis that PLA2R-H is the primary plasma membrane-associated form. To further validate this finding, we biotinylated the plasma membrane proteins and isolated them using streptavidin-conjugated magnetic beads. Consistent with our previous observation in Figure 1F, only PLA2R-H was precipitated (Figure 1G). Interestingly, there was a decreased level of PLA2R-H in PLA2R-Ab^+^ serum-treated samples, suggesting a reduction of PLA2R on the plasma membrane (Figure 1G). Similarly, flow cytometry analysis also demonstrated a significant decrease in surface PLA2R levels following treatment with PLA2R-Ab^+^ serum (Figure 1H-I), reinforcing that glycosylation status may guide plasma membrane localization of PLA2R and its recognition by pathogenic antibodies.

### Ectodomain shedding of PLA2R is not involved in PLA2R-Ab^+^ serum-induced downregulation of PLA2R

**Figure 2:**
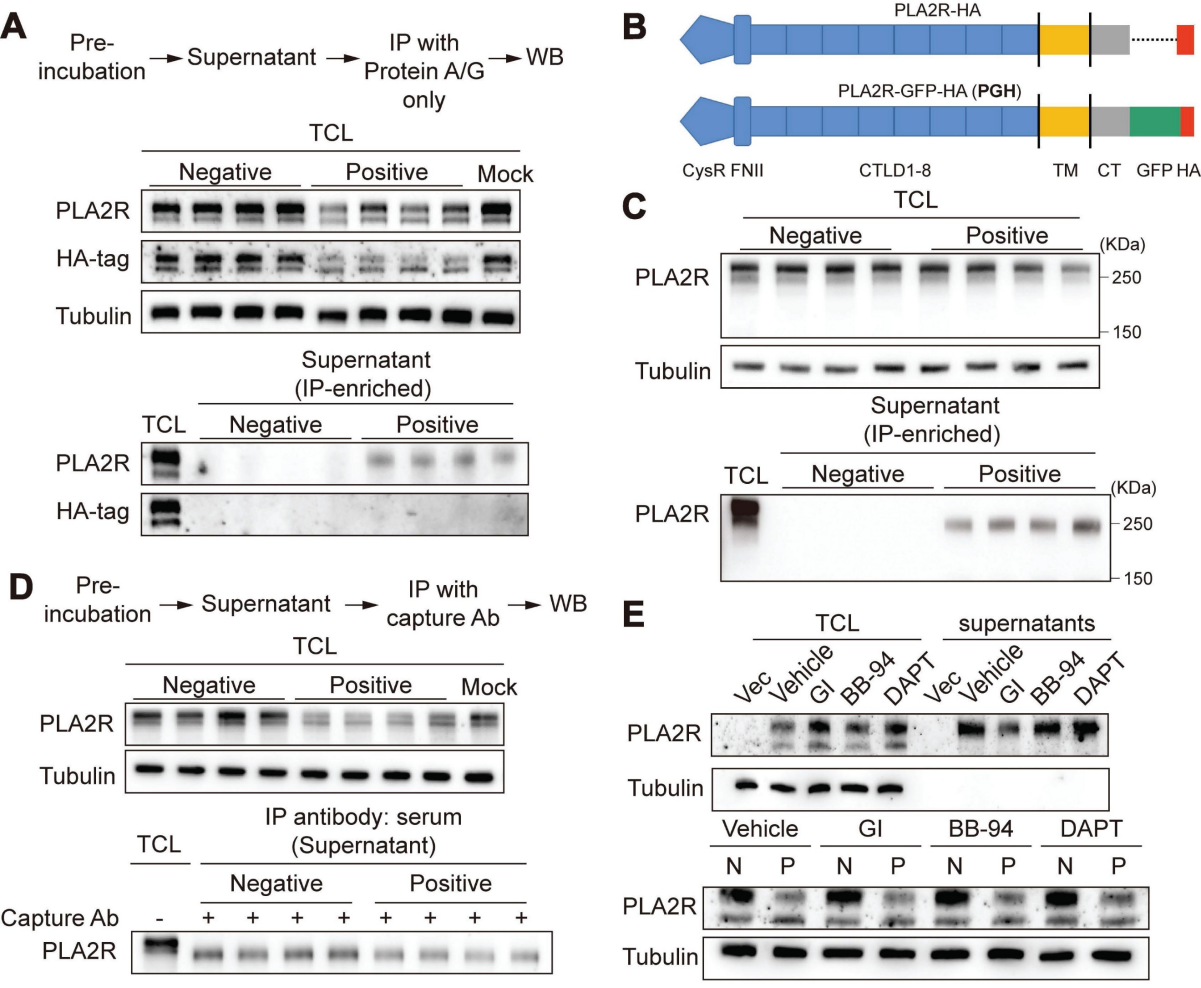
** Ectodomain shedding is not involved in PLA2R-Ab^+^ serum-induced downregulation of PLA2R.** (**A**) Supernatants from PLA2R-Jurkat cells were immunoprecipitated (IP) with Negative (n = 4) or Positive (n = 4) serum and then subjected to detection of PLA2R by western blot. TCL, total cell lysate; WB, western blot. (**B**) Schematic diagram of PLA2R-HA and PLA2R-GFP-HA (PGH) constructs. TM, transmembrane; CT, C-terminal. (**C**) Supernatants from PGH-Jurkat cells were IP with Negative (n = 4) or Positive (n = 4) serum and then subjected to detection of PLA2R by western blot. (**D**) PGH-Jurkat cells were pre-incubated for 6 h with Negative (n = 4) or Positive (n = 4) serum. Supernatants were collected and IP with Positive serum as capture Ab and then subjected to detection of PLA2R by western blot. N, negative; P, Positive. (**E**) Western blot analysis of PLA2R levels in PLA2R-Jurkat cells treated with Negative or Positive serum in the presence of the indicated inhibitors. Vehicle was the negative control. Tubulin was served as a loading control. Experiments were repeated 2-3 times with similar results.

Based on our earlier results, we hypothesized that the decrease in PLA2R expression observed in PLA2R-Ab^+^ serum-treated cells could be due to either increased release or degradation of PLA2R. To investigate whether PLA2R-Ab^+^ serum induced the release of PLA2R into the cell culture supernatants, we collected the cell culture supernatants from serum-treated cells. Initially, we could not detect PLA2R in either the raw cell culture supernatants or its ultrafiltrate concentrate, possibly due to our assay's low level of PLA2R or insufficient sensitivity. To enhance detection sensitivity, immune complexes formed in cell culture supernatants were immunoprecipitated using protein A/G agarose beads prior to analysis. With IP-based enrichment, we successfully detected soluble PLA2R in the cell culture supernatants (Figure 2A), confirming the presence of PLA2R released into the extracellular space. Conversely, soluble PLA2R was undetectable in cell culture supernatants from cells stimulated with negative serum. However, while the mouse PLA2R antibody (which targets the extracellular domain of PLA2R) detected the released form of PLA2R (Figure 2A, PLA2R panel), the HA-tag antibody (targeting the C-terminal intracellular domain) did not detect a corresponding band (Figure 2A, HA-tag panel). This suggests that the PLA2R released into the cell culture supernatants is likely a truncated form, consisting only of the ectodomain rather than the full-length PLA2R (FL-PLA2R). Previous studies have shown that the ectodomain of PLA2R can be cleaved by metalloproteinases and shed into the extracellular space, forming a soluble PLA2R (sPLA2R) lacking the transmembrane and intracellular domains 38, 39. To better visualize the size difference between s-PLA2R and FL-PLA2R, we added a green fluorescent protein (GFP) tag (approximately 26 kDa) to the C-terminus of PLA2R's intracellular domain, generating a construct referred to as PGH (PLA2R-GFP-HA) (Figure 2B). Following the IP of the cell culture supernatants, we observed PLA2R with a reduced size compared to FL-PLA2R in the TCL (Figure 2C), confirming that the PLA2R released into the cell culture supernatants is truncated. Next, we examined whether Positive serum can increase PLA2R shedding. To do this, we pre-incubated PLA2R-Jurkat cell with Negative or Positive serum for 6 h, and then detected PLA2R in cell culture supernatants after IP with the Positive serum. Unexpectedly, we observed that the Positive group showed less sPLA2R accumulation than the Negative control group (Figure 2D). Next, we investigated the involvement of ectodomain shedding in the PLA2R-Ab^+^ serum-induced downregulation of PLA2R expression. Previous studies suggest that PLA2R shedding is mediated by A Disintegrin And Metalloprotease (ADAM) 10/17 38. We used inhibitors for ADAM and matrix metalloproteinases (MMP) to explore this. Our results showed that the ADAM inhibitor GI254023X (GI) effectively reduced the accumulation of sPLA2R in the cell culture supernatants (Figure 2E, upper panel). In contrast, neither the MMP inhibitor batimastat (BB-94) nor the γ-secretase inhibitor DAPT had this effect (Figure 2E, upper panel). However, GI does not reverse the decreased PLA2R expression (Figure 2E, lower panel). These results suggest that while ectodomain shedding of PLA2R does occur, it is not the primary mechanism for downregulating PLA2R by PLA2R-Ab^+^ serum.

### PLA2R-Ab^+^ serum treatment triggered rapid internalization of PLA2R and translocation of PLA2R to lysosome

To investigate whether the PLA2R downregulation induced by PLA2R-Ab^+^ serum is linked to protein degradation, we first focused on internalization mechanisms. Like other members of the mannose receptor (MR) family, PLA2R can be internalized after ligand or antibody binding and subsequently transported to lysosomes via the endocytic pathway for degradation. We examined whether PLA2R antibodies trigger the internalization of PLA2R using PGH cells, where PLA2R and GFP are expressed as a fusion protein.

We generated a stable PGH-overexpressing MPC5 cell line (PGH-MPC5) for immunofluorescence assays. Western blot analysis confirmed that PLA2R-Ab^+^ serum stimulation led to the downregulation of PLA2R in PGH-MPC5 cells (Figure S2A). Next, we cultured PGH-MPC5 cells with the control or PLA2R-Ab^+^ serum and detected the localization of PLA2R-GFP and the PLA2R antibody. In control conditions (Negative serum), PLA2R was mostly diffusely distributed within the cells, with a small portion on the cell membrane (Figure S2B). Upon treatment with PLA2R-Ab^+^ serum, PLA2R aggregated in intracellular vesicular structures. Notably, we also observed PLA2R antibody (human IgG, hIgG) within cells only in the Positive group, and the hIgG signal nicely co-localized with PLA2R, suggesting that PLA2R and its antibody were internalized together as a complex (Figure S2B).

**Figure 3:**
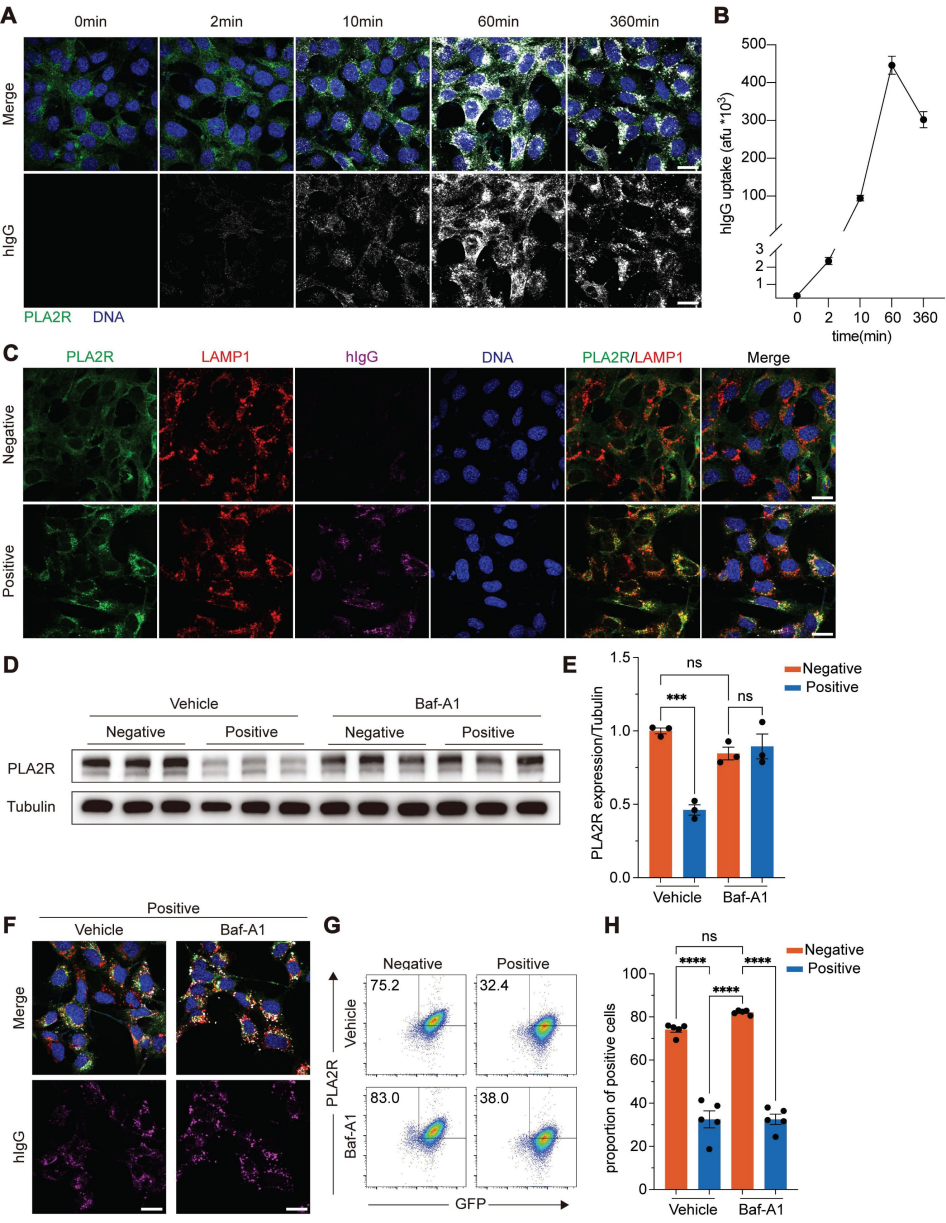
** PLA2R-Ab^+^ serum induces translocation of PLA2R to lysosome for degradation.** (**A**-**B**) Dynamics of intracellular human antibodies (hIgG) after treating PGH-MPC5 with Positive serum. Representative image and quantification are shown in **A** and **B**, respectively. Blue color represents DAPI staining for DNA and green color represents GFP for PLA2R. Afu, arbitrary fluorescence units. (**C**) Confocal analysis of PLA2R, LAMP1 and hIgG in PGH-MPC5 cells treated for 6 h with Negative or Positive serum. (**D**-**E**) Western blot analysis of PLA2R expression in PLA2R-MPC5 cells treated with Negative or Positive serum in the presence of negative control (Vehicle) or lysosome inhibitor Bafilomycin A1 (Baf-A1). Representative and quantification data are shown in **D** and **E**, respectively. (**F**) Confocal analysis of hIgG accumulation in PGH-MPC5 cells treated for 6 h with Negative or Positive serum in the absence or presence of Baf-A1. (**G**-**H**) Flow cytometry detection of PLA2R level on the surface of PLA2R-Jurkat cells treated with Negative (n = 5) or Positive (n = 5) serum, with or without Baf-A1. Representative and quantitative data are shown in **G** and **H**, respectively. Data are shown as the mean ± SEM. Experiments were repeated twice with similar results. Statistical significance was assessed using one-way ANOVA followed by Tukey multiple-comparison test (**E** and **H**). ns, non-significant; ***P < 0.001; ****P < 0.0001. Scale bars: 20 μm (**A**, **C** and **F**).

To further analyze the dynamics of PLA2R internalization, we tracked hIgG following PLA2R-Ab^+^ serum treatment. Immunofluorescence microscopy revealed a rapid appearance of intracellular hIgG signals, detectable as early as 2 min post-treatment, with a peak at 60 min, followed by a gradual decline (Figure 3A-B). Considering that the endocytic-lysosomal system is central to membrane protein degradation, we hypothesized that PLA2R antibodies could drive PLA2R internalization followed by its transfer to lysosomes. To test this, we labeled lysosomes with Lysotracker in PGH-MPC5 cells and exposed them to control or PLA2R-Ab^+^ serum. Our results showed a marked increase in the co-localization of PLA2R with lysosomes after PLA2R-Ab^+^ serum treatment, accompanied by an increase in lysosome size, compared to the control groups (Mock or Negative serum) (Figure S2C). To further probe the spatial relationship among PLA2R, PLA2R antibodies, and lysosomes, we used lysosome-associated membrane protein 1 (LAMP1) as a lysosomal marker. In the control groups, no significant co-localization of PLA2R with LAMP1 existed, and hIgG was absent. In contrast, in the Positive group, significant co-localization of PLA2R, PLA2R antibodies, and LAMP1 was observed (Figure 3C), suggesting lysosomal targeting of PLA2R-antibody complexes. Next, we tracked the dynamics of PLA2R and hIgG post-PLA2R-Ab^+^ serum stimulation. One hour after stimulation, both PLA2R and hIgG were internalized into the PGH-MPC5 cells but had not yet co-localized with LAMP1 (Figure S2D, 1 h). By 6 h post-stimulation, co-localization among PLA2R, PLA2R antibodies, and LAMP1 became evident (Figure S2D, 6 h), indicating that PLA2R is directed to lysosomes after internalization. Given our earlier findings, which showed that PLA2R levels begin to decrease approximately 6 h after PLA2R-Ab^+^ serum stimulation (Figure 1C), these results suggest that the lysosomal degradation pathway likely plays a critical role in the reduction of PLA2R levels triggered by PLA2R-Ab^+^ serum.

### Lysosomal activity was required for PLA2R-Ab^+^ serum-induced downregulation of PLA2R

To determine the pathway responsible for PLA2R degradation, we pre-treated cells with lysosomal inhibitors, bafilomycin A1 (Baf-A1) and chloroquine (CQ), and also used MG-132 to block the proteasome pathway. After pre-incubation with these inhibitors, cells were treated with control or PLA2R-Ab^+^ serum and analyzed for PLA2R expression. Our results showed that proteasome inhibition by MG-132 did not prevent the PLA2R degradation induced by PLA2R-Ab^+^ serum (Figure S3A-B). In contrast, Baf-A1 and CQ, which inhibit lysosomal activity, effectively restored PLA2R expression to levels comparable to the control group (Figure 3D-E and Figure S3A-B). These findings suggest that the lysosomal degradation pathway, rather than the proteasome pathway, is responsible for the PLA2R-Ab^+^ serum-induced downregulation of PLA2R.

Interestingly, treatment with Baf-A1 did not interfere with the internalization of PLA2R induced by PLA2R-Ab^+^ serum (Figure 3F). Flow cytometry analysis further confirmed that, even in the presence of Baf-A1, PLA2R-Ab^+^ serum continued to reduce surface PLA2R levels (Figure 3G-H). This indicates that while lysosomal inhibition can prevent the degradation of internalized PLA2R, it does not block the internalization process.

### Mutation of the endocytic domain blocked PLA2R-Ab^+^ serum-induced internalization of PLA2R and subsequent degradation

**Figure 4:**
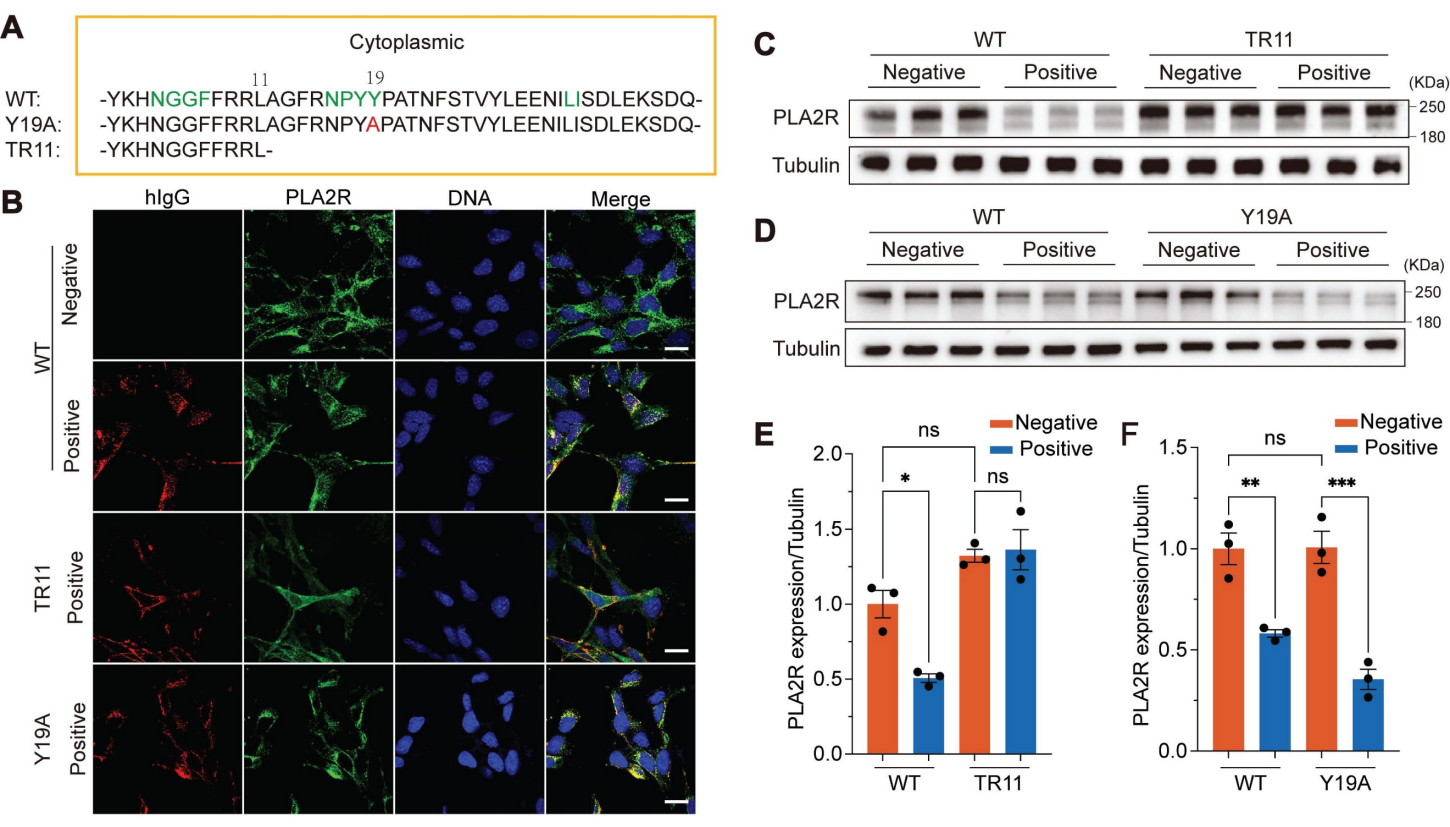
** Mutation of the endocytic domain blocks PLA2R-Ab^+^ serum-induced endocytosis and degradation of PLA2R.** (**A**) Schematic diagram for PLA2R mutants. The Arabic numerals above the sequence indicate the location of the residues located in the intracellular domain. Green-colored amino acids represent the endocytic sequence inferred by big data calculations, red represents the 19th amino acid mutation (PLA2R-Y19A, Y19A), and PLA2R-TR11 (TR11) refers to truncations starting from the 11th amino acid. WT, wild type; HA, HA-tag. (**B**) Confocal analysis for the localization of PLA2R and hIgG in WT- or TR11- or Y19A-expressing MPC5 cells treated with Negative or Positive serum. PLA2R and its mutants were detected by HA-tag antibody. (**C** and **E**) Western blot analysis of WT or TR11 levels in MPC5 cells treated for 6 h with Negative (n = 3) or Positive (n = 3) serum. Representative and quantitative data are shown in **C** and **E**, respectively. (**D** and **F**) Western blot analysis of WT or Y19A levels in MPC5 cells treated for 6 h with Negative (n = 3) or Positive (n = 3) serum. Representative and quantitative data are shown in **D** and **F**, respectively. Data are shown as the mean ± SEM. Experiments were repeated twice with similar results. Statistical significance was assessed using one-way ANOVA followed by Tukey multiple-comparison test (**E** and **F**). ns, non-significant; *P < 0.05, **P < 0.01, ***P < 0.001. Scale bars: 20 μm (**B**).

To investigate whether the endocytic domain of PLA2R is essential for PLA2R-Ab^+^ serum-induced internalization and subsequent degradation, we generated two PLA2R mutants: Y19A, in which the 19th tyrosine (Y) in the C-terminal intracellular region was mutated to alanine (A), and TR11, which retained only the first 11 amino acids of the intracellular region (Figure 4A). According to the previous report, these mutations are designed to impair PLA2R endocytosis 24. To assess the effects of these mutations on PLA2R internalization, we incubated the cells with PLA2R-Ab^+^ serum and monitored the localization of the two mutants using immunofluorescence microscopy. Our results showed that wild-type (WT) PLA2R or the Y19A mutant was internalized alongside human IgG (hIgG) (Figure 4B). In contrast, no internalization occurred in cells expressing the TR11 mutant, where both PLA2R (TR11) and hIgG remained on the plasma membrane (Figure 4B). This indicates that although the TR11 mutant retains its ability to bind to PLA2R antibodies, it blocks internalization induced by PLA2R-Ab^+^ serum. We then examined whether blocking internalization would interfere with PLA2R degradation induced by PLA2R-Ab^+^ serum. Western blot analysis showed that PLA2R-Ab^+^ serum reduced the expression levels of WT-PLA2R but had no effect on the TR11 mutant (Figure 4C and Figure 4E). As expected, the Y19A mutation did not prevent internalization, and PLA2R levels decreased similarly to the WT group following PLA2R-Ab^+^ serum treatment (Figure 4D and Figure 4F). These findings suggest that PLA2R antibodies in PMN patient serum promote PLA2R internalization, which is crucial for its subsequent degradation. The inability of the TR11 mutant to undergo internalization confirms that endocytosis is a prerequisite for PLA2R degradation.

### Dynamin-dependent Clathrin-mediated endocytosis is required for the internalization of PLA2R upon PLA2R-Ab^+^ serum treatment

**Figure 5:**
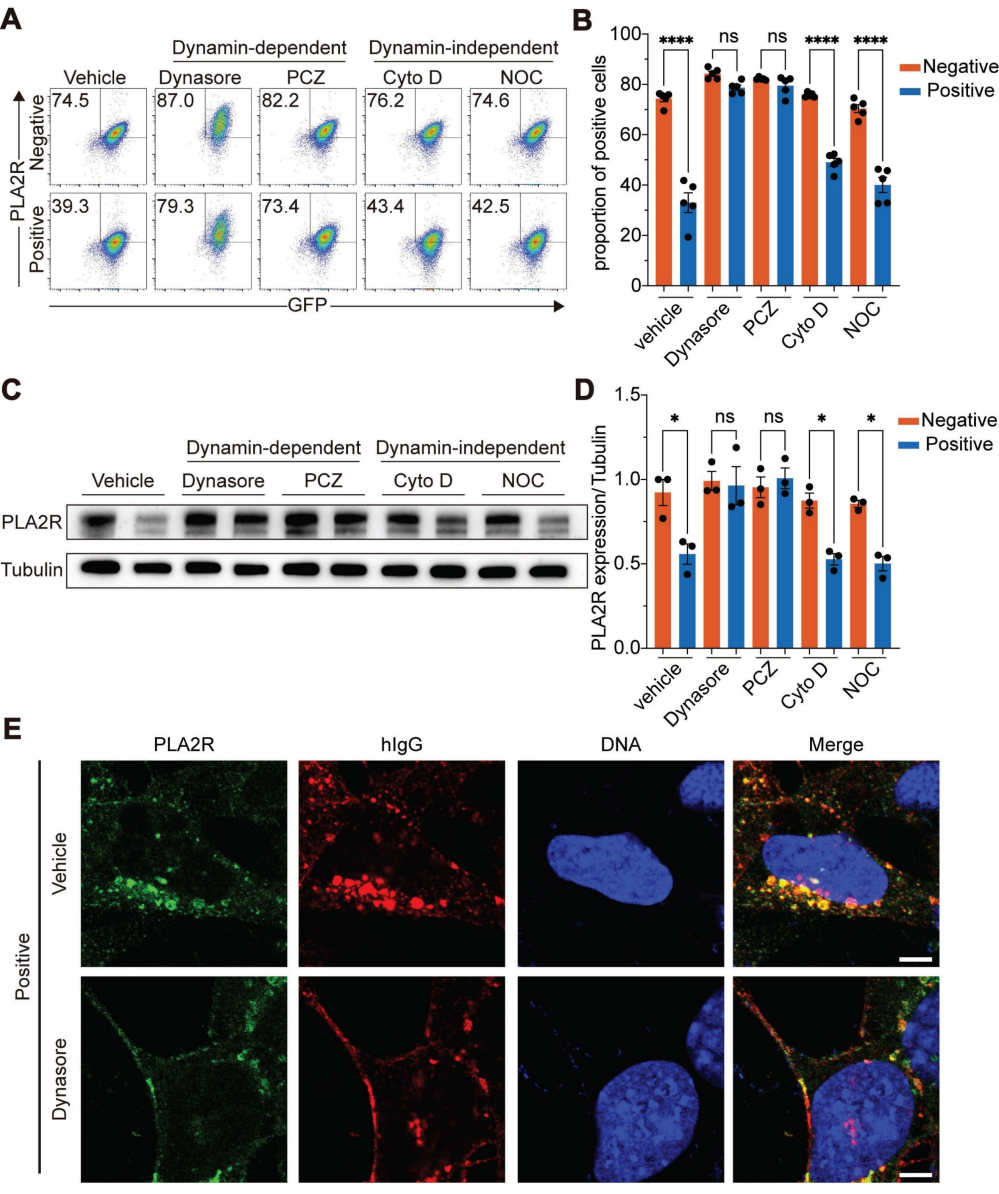
** PLA2R-Ab^+^ serum-induced endocytosis is dependent on CME pathway.** (**A**-**B**) Flow cytometry detection of PLA2R level on the surface of PLA2R-Jurkat cells treated with Negative (n = 5) or Positive (n = 5) serum in the presence of negative control (Vehicle) or the indicated inhibitors. Representative and quantitative data are shown in **A** and **B**, respectively. PCZ, prochlorperazine; Cyto D, CytochalasinD; NOC, nocodazole. (**C**-**D**) Western blot analysis of PLA2R expression in PGH-MPC5 cells treated with Negative (n = 3) or Positive serum (n = 3) in the presence of indicated inhibitors. Representative and quantification data are shown in **C** and **D**, respectively. (**E**) Confocal analysis of PLA2R and hIgG accumulation in PGH-MPC5 cells treated for 6 h with Positive serum in the absence or presence of Dynasore. Data are shown as the mean ± SEM. Experiments were repeated twice with similar results. Statistical significance was assessed using one-way ANOVA followed by Tukey multiple-comparison test (**B** and **D**). ns, non-significant; *P < 0.05, ****P < 0.0001. Scale bars: 5 μm (**E**).

We tested the role of various endocytic pathways to determine the mechanism of PLA2R internalization triggered by PLA2R antibodies. We treated cells with prochlorperazine (PCZ) to inhibit clathrin-mediated endocytosis (CME), dynasore to block dynamin-dependent endocytosis, CytochalasinD (Cyto D) to inhibit aggregation-dependent endocytosis (ADE), and nocodazole (NOC) to block micropinocytosis 38, 40-44. Flow cytometry was used to measure surface PLA2R levels as an indicator of endocytosis. The results showed that, compared to the control group (vehicle), both PCZ and dynasore efficiently blocked the reduction of surface PLA2R induced by PLA2R-Ab^+^ serum (Figure 5A-B). Furthermore, similar to the TR11 mutation, inhibiting internalization with either dynasore or PCZ prevented the PLA2R degradation triggered by PLA2R-Ab^+^ serum (Figure 5C-D, and Figure S3C-F). In contrast, groups treated with Cyto D or NOC, which did not impact internalization, still exhibited a decrease in PLA2R level after PLA2R-Ab^+^ serum treatment (Figure 5C-D). Immunofluorescence analysis revealed that dynasore treatment caused PLA2R to accumulate on the cell membrane in both the Negative and Positive groups (Figure 5E). Importantly, in the Positive group, most antibodies (hIgG) remained on the cell surface without entering the cells (Figure 5E). These findings suggest that the internalization of PLA2R upon binding to PLA2R antibodies occurs through a dynamin-dependent CME pathway. Blocking this pathway with either dynasore or PCZ prevents the internalization and subsequent degradation of PLA2R.

### Recombinant PLA2R antibody caused lysosome-dependent degradation of PLA2R

**Figure 6:**
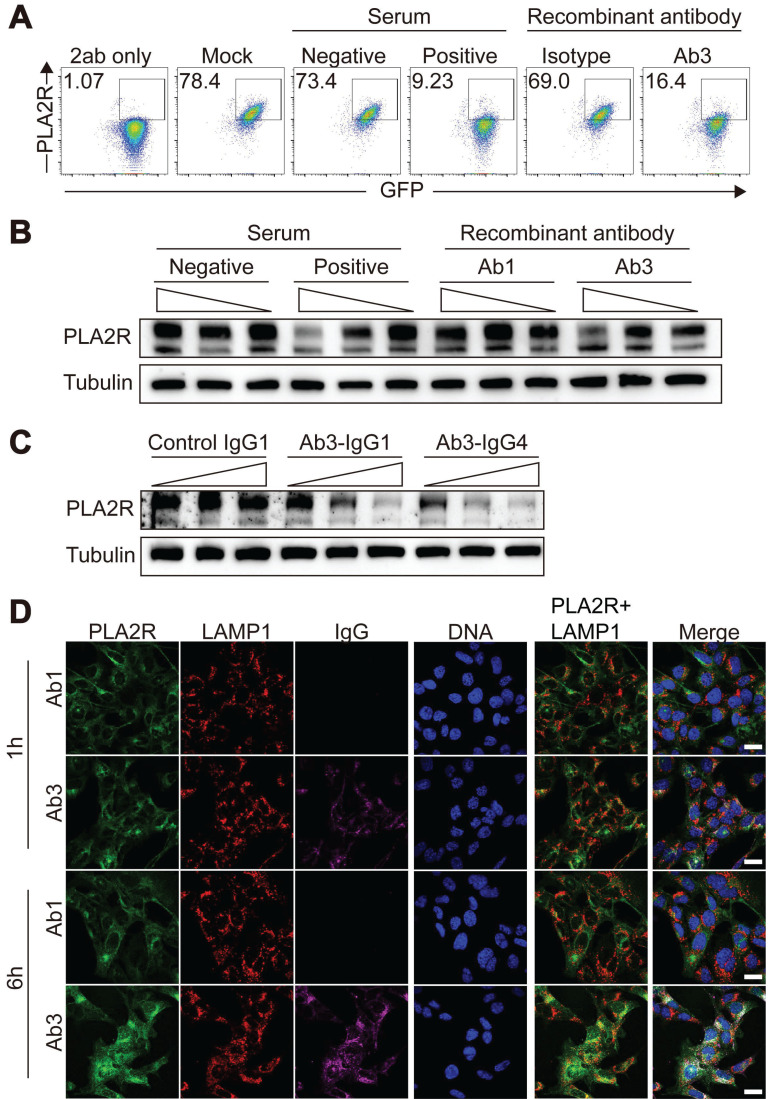
** Recombinant PLA2R antibody induces PLA2R endocytosis and degradation.** (**A**) Flow cytometry detection of PLA2R on the surface of PLA2R-Jurkat cells treated with Negative or Positive serum or recombinant PLA2R antibodies. Ab1 was not reactive to PLA2R and used as a negative control. (**B**) Western blot analysis of PLA2R expression in PLA2R-Jurkat cells treated for 6 h with serial dilution of Positive serum (5, 1, and 0.5 RU/mL, from high to low) or an equal volume of Negative serum as control, as well as Ab1-hIgG1 (10, 2, and 0.4 μg/mL, from high to low) or Ab3-hIgG1 (10, 2, and 0.4 μg/mL, from high to low). (**C**) Western blot analysis of PLA2R expression in PLA2R-Jurkat cells treated for 6 h with serial dilution of Ab3-hIgG1 (0.4, 2, 10 μg/mL, from low to high) or Ab3-hIgG4 (0.4, 2, 10 μg/mL, from low to high). Ab1-IgG1 was used as a negative control. (**D**) Confocal analysis of the subcellular localization of PLA2R-GFP (green), LAMP1 (red), and hIgG (purple) in PGH-MPC5 cells cultured for 1 h or 6 h in the presence of Ab1 or Ab3. Scale bars: 20 μm (**D**). Experiments were repeated twice with similar results.

To ensure that the effects observed with PLA2R-Ab⁺ serum were not due to non-specific activities, we employed a purified recombinant anti-PLA2R antibody produced in-house, generated according to previously described protocols 45, and we selected a specific antibody clone (Ab3) that targeted the CysR domain of PLA2R (Figure S4A-C). Another clone (Ab1) that showed no recognition to PLA2R was used as the negative control. Our results showed that Ab3-IgG1 mimicked the effects of PLA2R-Ab^+^ serum, as it led to a reduction in surface PLA2R levels (Figure 6A), a decrease in total PLA2R expression (Figure 6B), and induced a time-dependent internalization of PLA2R into lysosomes (Figure 6C). Since PLA2R autoantibodies in serum samples from PMN patients are mainly IgG4 5, we sought to determine if IgG subclass will affect antibody-induced downregulation of PLA2R. As shown in Figure 6D, both Ab3-IgG1 and Ab3-IgG4 induced similar downregulation of PLA2R, suggesting that antibody recognition but not the IgG subclass is crucial for this process. These results collectively indicate that PLA2R has the intrinsic characteristic of being internalized upon antibody binding. This binding initiates the endocytosis of PLA2R, followed by its transport to lysosomes for degradation.

### PLA2R-Ab^+^ serum-induced PLA2R internalization boosts antigen presentation

**Figure 7:**
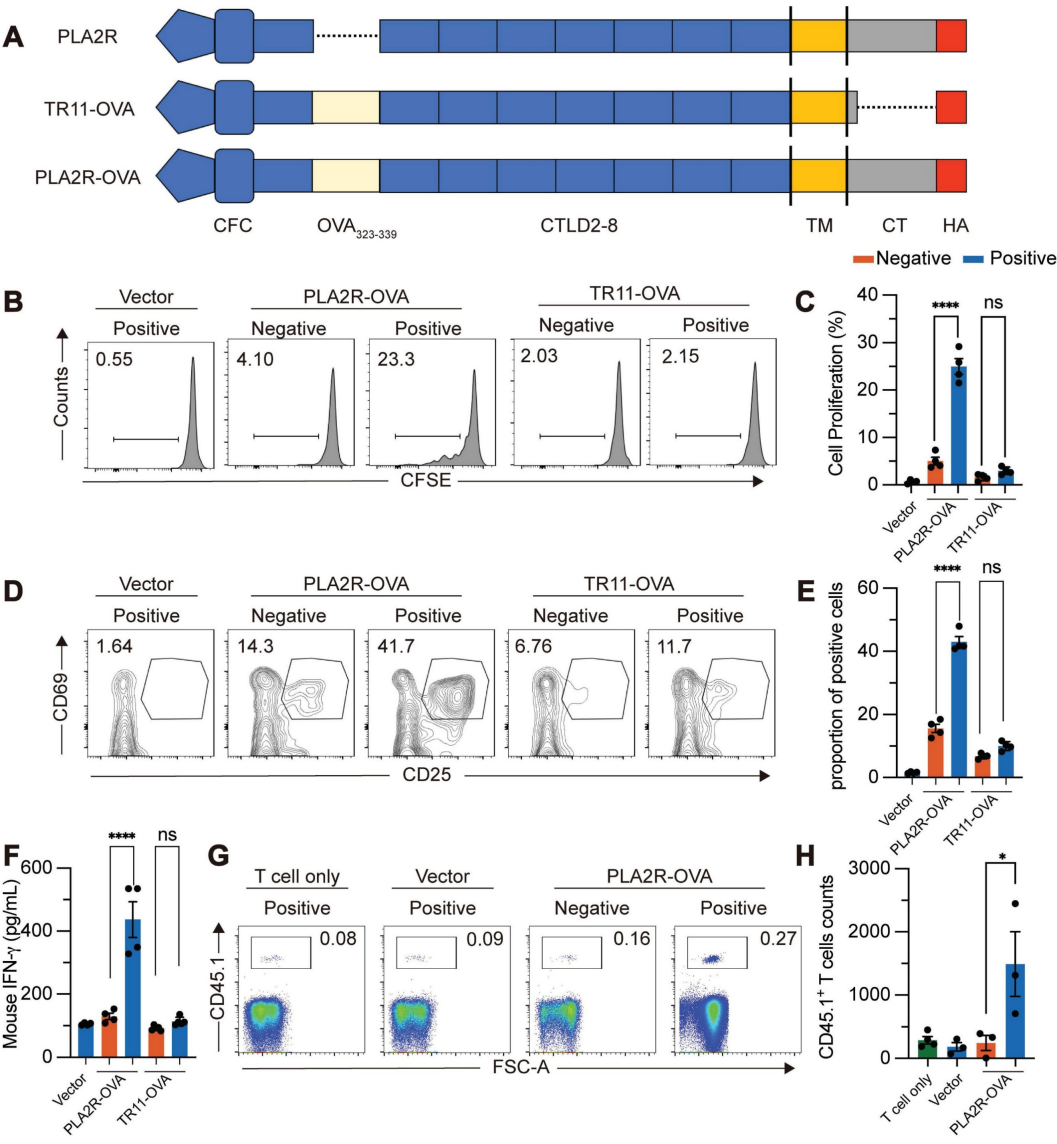
** PLA2R-Ab^+^ serum-induced PLA2R endocytosis promotes antigen presentation and activation of antigen-specific CD4^+^ T cells.** OTII CD4^+^ T cells were incubated with Negative (n = 4) or Positive (n = 4) serum-treated PLA2R-OVA-expressing BMDCs. Three days after incubation, proliferation (**B** and **C**), activation (**D** and **E**) and cytokine production (**F**) of CD4^+^ T cells were measured. BMDC, bone-marrow derived dendritic cells. (**A**) Schematic diagram of PLA2R-OVA construction. OVA_323-339_ was inserted between N-terminal CFC region (CysR, FnII and CTLD1) and CTLD2 domain. (**B** and **C**) Flow cytometry analysis of the proliferation levels. Numbers represent frequencies of the proliferated cells based on CFSE-dilution. CFSE, carboxyfluorescein succinimidyl ester. Representative and quantitative data are presented in **B** and **C**, respectively. Vector, empty lentivirus. PLA2R-OVA, PLA2R-OVA-expressing lentivirus. (**D** and **E**) Flow cytometry analysis of T cell activation measuring expression of CD25 and CD69. Numbers indicate the proportion of CD25 and CD69 double-positive cells. Representative and quantitative data are presented in **D** and **E**, respectively. Data are shown as the mean ± SEM. (**F**) Level of IFN-γ were determined by ELISA. (**G** and **H**) Flow cytometry analysis of transferred cells (CD45.1⁺) in inguinal lymph node. Frequencies of transferred cells in total lymph node cells are shown in G, and absolute numbers of transferred cells are quantified in H. Experiments were repeated 3 times with similar results. Statistical significance was assessed using one-way ANOVA followed by Tukey multiple-comparison test (**D**-**G**). ns, non-significant; ***P < 0.001, ****P < 0.0001.

Lysosomes serve as organelles for protein degradation and are the sites where MHCII molecules in APCs assemble into MHCII/peptide complexes 46, 47. The MHCII/peptide complexes are displayed on the plasma membrane to activate antigen-specific CD4^+^ T cells. We explored whether PLA2R-Ab^+^ serum-induced internalization of PLA2R enhances MHCII-mediated antigen presentation, potentially amplifying PLA2R-specific CD4^+^ T cell responses. Since no PLA2R-specific CD4^+^ T cell clones are available, we constructed a fusion protein combining PLA2R with the widely recognized OVA_323-339_ epitope from ovalbumin (PLA2R-OVA) (Figure 7A). This allowed us to use OT-II CD4^+^ T cells, which specifically respond to the OVA_323-339_/MHCII complex presented by APCs, as a model system. First, we confirmed that the PLA2R-OVA fusion protein could be internalized, transported to lysosomes, and degraded after stimulation with PLA2R-Ab^+^ serum (Figure S5A-B). This confirmed that the addition of the OVA_323-339_ sequence did not disrupt the normal function of PLA2R. Next, we tested whether PLA2R antibody-induced degradation enhances MHCII-dependent antigen presentation. We used lentivirus to deliver PLA2R-OVA into mouse bone marrow-derived dendritic cells (BMDCs), which were then cocultured with naïve OT-II CD4^+^ T cells. Flow cytometry revealed that PLA2R-OVA-expressing BMDCs stimulated with PLA2R Ab⁺ serum significantly enhanced OT-II CD4⁺ T cell activation compared to negative controls (Figure 7B-F). Specifically, serum antibodies increased T cell proliferation (Figure 7B-C), upregulated CD25 and CD69 expression (Figure 7D-E), and augmented IFN-γ production (Figure 7F). On the contrary, TR11-OVA-expressing BMDCs were incompetent for boost T cell response upon serum antibody treatment (Figure 7B-F), confirming antibody-induced PLA2R internalization is essential for PLA2R-mediated antigen presentation. To assess whether this occurs *in vivo*, we transferred CD45.1⁺ CD4⁺ OT-II T cells into CD45.2⁺ recipient mice, followed by administration of PLA2R-OVA or PLA2R-TR11-OVA-expressing BMDCs pretreated with PMN patient or control sera. Consistent with the *in vitro* findings, PLAR-OVA-expressing BMDCs significantly promoted OT-II CD4 T cell expansion *in vivo* (Fig. 7G-H).

### PLA2R is expressed in human lymph node

Although podocytes express high levels of PLA2R and the antigen-presenting molecule HLA-DR, the absence of CD4⁺ T cells in the glomeruli of PMN patients makes it unlikely that podocytes serve as the primary antigen-presenting cells responsible for activating PLA2R-specific T cells. To identify potential APCs that express PLA2R, we queried the Human Protein Atlas database. Based on the data from The Human Protein Atlas (https://www.proteinatlas.org/ENSG00000153246-PLA2R1/tissue), PLA2R is expressed not only in the kidney but also in other tissues, including the lung, skeletal muscle, testes and lymph node. Given that lymph node serves as the primary site for antigen presentation where structural compartmentalization organizes antigen-presenting cells (APCs), T cells, and B cells into specialized microenvironments 48, 49, we further investigated PLA2R expression in lymph node. PCR and immunohistochemistry analyses confirmed the presence of PLA2R in human lymph nodes (Figure S6A-B). Immunofluorescence staining revealed partial co-localization of PLA2R with CD31, an endothelial cell marker (Figure S6C). Furthermore, flow cytometry analysis revealed that while PLA2R expression was undetectable in CD45⁺ cells constituting the majority of PBMCs and lymph node cellularity (Figure S6D), PLA2R was specifically expressed in the lymph node stromal cells (CD45^-^), particularly in fibroblastic reticular cells (FRC) and lymphatic endothelial cells (LEC) (Figure S6E).

**Figure 8:**
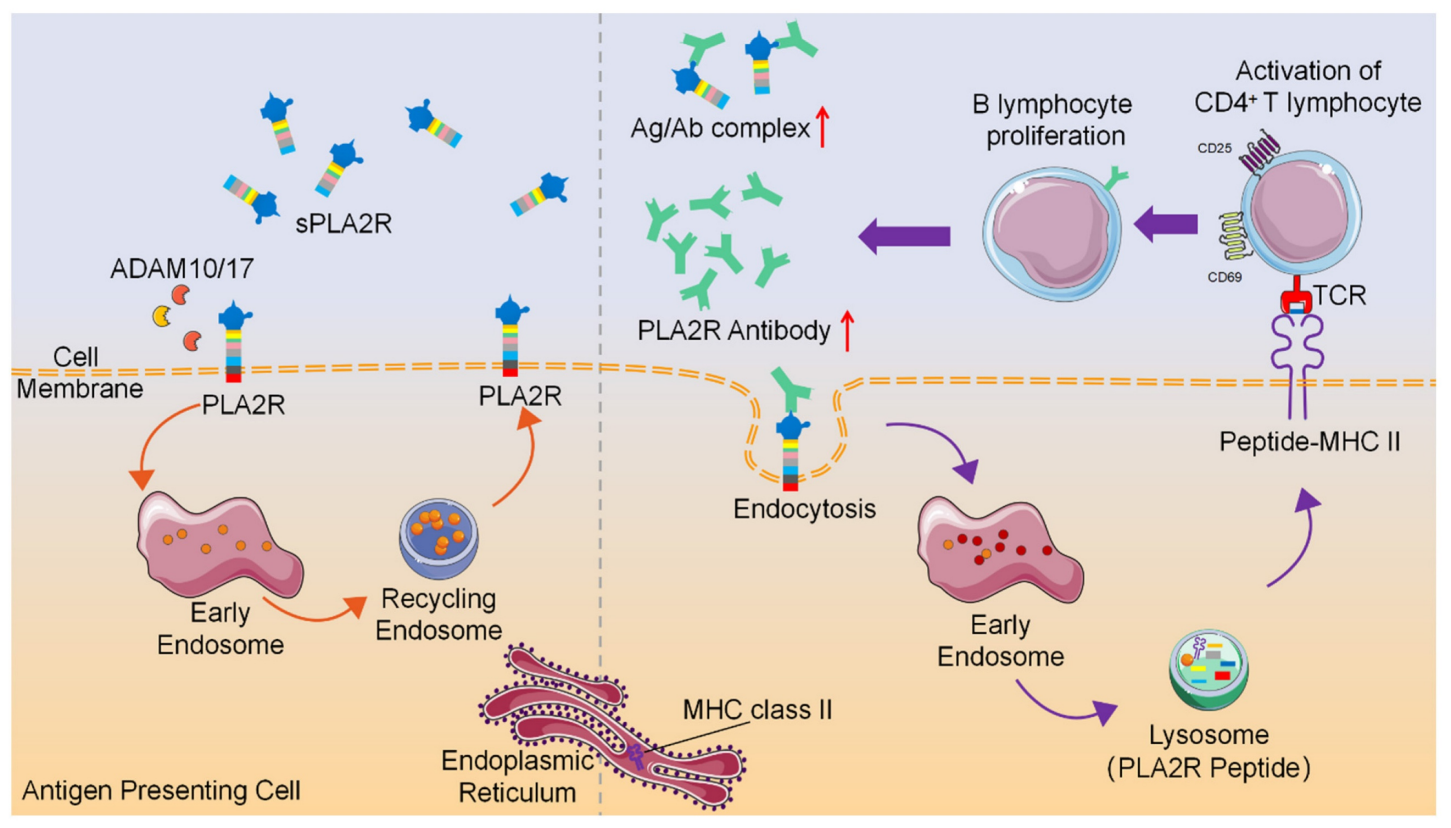
** Schematic diagram of PLA2R antibody-induced internalization of PLA2R to facilitate antigen presentation to CD4^+^ T cells**.

Collectively, these findings indicate that PLA2R antibodies promote internalization and lysosomal degradation of PLA2R, a process that, when occurring in APCs, may facilitate peptide loading onto MHC-II molecules and enhance antigen-specific CD4⁺ T cell activation (Figure 8). This study provides evidence that antibody-induced PLA2R internalization boosts antigen presentation, potentially contributing to the amplification of autoimmune responses in PMN patients.

## Discussion

The discovery of PLA2R as the autoantigen in PMN represents a significant milestone in the field 5. This finding not only offers a sensitive and specific diagnostic tool for clinical practice but also reveals the autoimmune nature of PMN. While nearly twenty autoantigens associated with PMN have been identified, PLA2R antibodies remain prevalent in approximately 70% of PMN patients 50. Therefore, understanding the mechanisms behind generating PLA2R antibodies remains a central focus in PMN research. Our study found that PLA2R can be internalized upon stimulation with PLA2R-Ab^+^ serum or recombinant anti-PLA2R antibodies. This internalization occurs rapidly and depends on the CME pathway, requiring a consensus motif located in the cytoplasmic domain of PLA2R. The internalized cargo is subsequently directed to lysosomes for degradation. This process in APCs facilitates the loading of PLA2R-derived peptides onto MHCII molecules in lysosomes, thereby activating antigen-specific CD4^+^ T cells. Our findings suggest that PLA2R-mediated endocytosis may be crucial in developing anti-PLA2R autoimmunity in PMN.

The physiological role of PLA2R in PMN is incompletely understood. One study suggests that PLA2R participates in sPLA2 IB-induced apoptosis in human podocytes via the p53 pathway 51. Another study indicates that PLA2R mediates the adhesion of podocytes to GBM by interacting with collagen IV, likely through the FnII domain 52. PLA2R antibodies could block this interaction, potentially leading to podocyte detachment from the GBM 52. Given rodents lack PLA2R expression on podocytes yet maintain normal podocyte function, PLA2R may not be essential for podocyte function in a resting state, at least in rodents. Recently, Haddad et al. uncovered a novel mechanism underlying podocyte damage in PMN 37. They discovered that, in the presence of complement, PLA2R antibodies, especially the galactose-deficient IgG4 subtype, bind to PLA2R and lead to the degradation of two critical podocyte proteins, synaptopodin and NEPH1, explaining how PLA2R antibodies may induce podocyte injury 37. Our work demonstrated that PLA2R can function as an endocytic receptor and can be internalized upon PLA2R antibody treatment. Moreover, we showed that recombinant PLA2R antibodies of either the IgG1 or IgG4 subtype can trigger the internalization of PLA2R, highlighting that antibody recognition but not the IgG subtype is crucial for this process (Figure 6C). Consistent with the previous report 37, we did not observe any effect of PLA2R antibody on the apoptosis of PLA2R-expressing murine podocyte cells (Figure S1K).

Endocytosis is a finely regulated process that can be classified into CME and clathrin-independent endocytosis (CIE) 53. CME is the primary endocytic pathway in eukaryotic cells 54, 55. Each member of the MR family contains consensus motifs within their cytoplasmic domain that facilitate recruitment to clathrin-coated pits, enabling continuous endocytosis of exogenous cargo 25. Studies have shown that deleting specific amino acids or mutating certain residues in the intracellular domain can inhibit PLA2R endocytosis 25. In our experiments, PLA2R antibodies were detected intracellularly shortly after being introduced to the PLA2R-expressing cells, indicating a potential CME mechanism. Additionally, specific CME inhibitors such as dynasore or PCZ effectively blocked PLA2R endocytosis induced by PLA2R antibodies, confirming that the pathway for PLA2R-mediated endocytosis is indeed CME. Given temporary inhibition of endocytosis using PCZ has been proposed for various clinical applications, including viral infections, chronic kidney disease, and cancer 56-59, blocking the CME pathway with PCZ to inhibit PLA2R endocytosis may disrupt the functional effects of PLA2R antibodies, offering a potential intervention strategy for PMN.

It has long been established that soluble PLA2R is present in both human and mouse serum 39, 60, likely due to the constitutive shedding of its extracellular region 38. Other members of the MR family, such as MR and Endo180, can also be cleaved by MMPs or ADAMs, highlighting the common feature of ectodomain shedding within this family 61, 62. Accordingly, researchers have proposed that autoantibody binding to PLA2R initiates the in-situ formation of PLA2R-antibody immune complexes (ICs) and their subsequent shedding, leading to the accumulation of ICs in the subepithelial space of the GBM. Indeed, we found that treatment with PLA2R-Ab^+^ serum decreased the levels of PLA2R in the cell supernatant (Figure 2D), which may be attributed to the reduced level of PLA2R in the antibody-treated cells. This suggests that PLA2R antibodies may not actively regulate the shedding of PLA2R. Several studies have shown enhanced staining of PLA2R in glomeruli of most PLA2R-Ab^+^ patients compared to PLA2R-Ab-negative controls 63-66. Since this increased level of PLA2R was not due to altered mRNA regulation 64, the reason for the enhancement remains unclear. Based on our results and those of others 38, ICs in PLA2R-associated MN may form when circulating PLA2R antibodies traverse the GBM and bind soluble PLA2R shed by podocytes. Their size likely prevents urinary excretion, resulting in retention and enhanced PLA2R signal in the subepithelial space of glomeruli. Approximately 10% of PLA2R-Ab^+^ PMN patients exhibit negative detection of PLA2R in the glomeruli 63-67. As these patients exhibit unexpectedly higher levels of PLA2R antibody titers and poorer prognoses than their double-positive counterparts 65, 67, the absence of PLA2R in this group may result from a failure to accumulate ICs in the GBM. This could be due to severe damage to the filtration system or reduced levels of soluble PLA2R caused by persistent PLA2R-antibody-induced downregulation of PLA2R.

MHCII molecules are type I transmembrane proteins, which are synthesized in the endoplasmic reticulum and primarily localize within lysosomes. MHCII molecules share a similar structure, consisting of homodimerized alpha and beta chains that can form a luminal domain at the C-terminus, which includes the peptide-binding groove 68. In the endoplasmic reticulum, the peptide-binding groove of MHCII associates with an invariant chain (Ii), forming an αβIi multimeric complex 69. The binding of Ii spatially occupies the peptide-binding groove, preventing premature peptide binding. The MHCII multimeric complex is transported to lysosomes via clathrin-coated endocytic vesicles 69. Lysosomes not only can digest intracellular and extracellular macromolecules but are also where MHCII recognizes antigens and processes them to form antigen peptide/MHCII complexes 69. Our research demonstrates that PLA2R-Ab^+^ serum can enhance PLA2R transport to lysosomes for MHCII-mediated antigen presentation, potentially contributing to a positive feedback loop that gradually amplifies PLA2R-specific CD4^+^ T-cell responses, further increasing PLA2R antibody levels.

Based on the genetic evidence and the essential role of CD4^+^ T cells in regulating B cell function, the emergence and activation of PLA2R-specific CD4^+^ T cells likely play a significant role in autoantibody production in PMN. Although human podocytes express PLA2R and possess antigen-presenting capabilities, glomeruli in PMN patients typically do not show infiltration of inflammatory cells such as CD4^+^ T cells, which essentially rules out podocytes as the primary antigen-presenting cells responsible for initiating the PLA2R-specific T cell responses. PMN recurrence in kidney transplant recipients exceeds 40%, indicating that extrarenal factors contribute to disease development 70. Studies have shown a positive correlation between increased PM2.5 levels and PMN incidence 71, 72, suggesting that PM2.5-induced lung inflammation may trigger or amplify PLA2R autoimmune responses. Furthermore, case reports indicate that patients with ANCA-associated glomerulonephritis or those with hepatitis B or C infections may upregulate PLA2R expression due to localized inflammatory environments, potentially contributing to PLA2R antibody production 73-75. Interestingly, PLA2R is detectable in human lymph nodes (Figure S6), the principal site of antigen presentation. However, whether PLA2R expression in lymph nodes contributes to PLA2R-specific T cell responses in PMN remains to be determined.

Our study has several limitations. First, although we identified specific human APCs expressing PLA2R (LEC and FRC) within lymph nodes, lack of PLA2R-specific CD4^+^ T cell clones limit our ability to demonstrate that antibody-induced PLA2R endocytosis self-reinforcing PLA2R-specific CD4^+^ T cell responses in humans. Future studies could utilize techniques such as ELISpot or MHCII-tetramer assays to detect PLA2R-specific T-cell responses. Second, antibody-induced downregulation of PLA2R may enhance autoimmunity through additional mechanisms. RNA-seq data from ImmGen indicate that PLA2R is expressed in medullary thymic epithelial cells (mTECs), a type of unique stromal cells in the thymus responsible for establishing central tolerance in T cells. However, whether PLA2R expression on mTECs contributes to PLA2R-specific T cell responses warrants further investigation.

In summary, our study demonstrates that PLA2R antibodies in the serum of PMN patients bind to PLA2R, inducing PLA2R internalization through CME pathway and its subsequent transport to late endosomes for lysosomal degradation. During this process, PLA2R degradation enhances its efficiency as an antigen, leading to the activation of antigen-specific CD4^+^ T cells. Although the clinical treatment of PMN remains challenging, we hope that our research provides new insights into the prevention and treatment of PMN, paving the way for future clinical applications in its diagnosis and management.

## Materials and Methods

*Sex as a biological variable.* Our study examined serum samples from male and female subjects, and similar findings are reported for both sexes.

*Human samples*. Human blood and serum samples were collected from patients with membranous nephropathy or with other glomerular disorders. The samples were assigned codes to render them anonymous. Serum was incubated in a 56 °C for 30 min to inactivate complement. The study was approved by the Ethics Committee of Nanfang Hospital, Southern Medical University, and written informed consent was obtained from all participants before collection of peripheral whole blood samples (Approval No. NFEC-202205-K20). Human lymph node were obtained as discarded tissue in non-malignancy surgeries from the Nanfang Hospital (Approval No. NFEC-2019-263).

*Mice*. C57BL/6 mice were obtained from the Laboratory Animal Centre of Southern Medical University (Guangzhou, China). OT-II mcie were purchased from The Jackson Laboratory. Animal studies were conducted at Southern Medical University and were approved by the University Committee on Use and Care of Animals (UCUCA) (Approval No. SMUL202502004).

*PLA2R antibody titer determination and treatment*. PLA2R antibodies in the serum samples were detected using the EUROIMMUN ELISA test as part of the clinical assessment of the patients. According to the manufacturer's guidelines, titers below 14 RU/mL are considered negative, while values ≥ 20 RU/mL are positive. In this study, serum samples with PLA2R antibody levels < 2 RU/mL were classified as PLA2R antibody negative (Negative), while values for PLA2R antibody-positive (PLA2R-Ab^+^) sera ranged from 366.4 to 2583 RU/mL. For cell treatments, PLA2R-Ab^+^ serum samples were normalized to a working concentration of 5 RU/mL.

*Reagents*. RPMI-1640 (C11875500BT), DMEM (C11995500BT), FBS (10099141C), penicillin/streptomycinwere (15140122), HEPES (15630080), pyruvate (25030081), b-ME (21985023) and Sulfo-NHS-SS-biotin (21336) were purchased from Thermo Fisher Scientific; CFSE (21888), Chloroquine (CQ, C6628, 100 μM), Prochlorperazine (PCZ, P9178, 5 μM) and DMSO (D2650) were purchased from Sigma-Aldrich. MG-132(2194S, 10 μM) were purchased from Cell Signaling Technology. PNGase-F (P0705S), XbaI (R0145S) and EcoRI (R3101S) were purchased from New England Biolabs; Bafilomycin A1 (Baf-A1, S1413, 200 nM), GI254023X (GI, S8660, 550 nM), DAPT (S2215, 10 μM) and Batimastat (BB-94, S7155, 1 μM) were purchased from Selleck Chemicals; Cocktail (HY-K0010), Nocodazole(HY-13520, 10 μM), Dynasore (HY-13863, 90 μM), Cytochalasin D (HY-N6682, 30 μM) were obtained from MedChemExpress. CD19 magnetic beads (130-050-301) were from Miltenyi Biotec. Human Fc block (422302) was from BD Biosciences. GM-CSF (20 ng/mL), recombinant PLA2R and recombinant THSD7A were provided by Epoto Biotech. Streptavidin Magnetic Beads (P2151) and Lyso-Tracker Red (C1046) were purchased from Beyotime; Protein A/G Agarose beads (sc-2003) were from Santa Cruz. Antibodies used in this study: PLA2R (ab211490, Abcam); HA-tag (3724S, CST); CD107a (LAMP-1) (14-1071-82, Thermo Fisher Scientific); alpha-Tubulin (66031-1, Proteintech); GAPDH (60004-1, Proteintech); PerCP/Cy5.5 anti-mouse CD4 (100540, Biolegend); APC anti-mouse CD25 (102012, Biolegend); PE/Cy7 anti-mouse CD69 (104512, Biolegend); PerCP/Cy5.5 anti-human CD45 (304028, biolegend); FITC anti-human CD31 (303103, biolegend); PE anti-human Podoplanin (PDPN, 12-9381-42, eBioscience); BV421 anti-HLA-DR (307635, biolegend); eFluor 780 Viability Dye (65-0865-18, eBioscience); FITC anti-human CD20 (555622, BD); BV421 anti-human IgG (562581, BD); PE/Cy7 anti-human CD27 (560609, BD).

*Cell culture*. Jurkat, HEK293T and NIH3T3 cell lines were purchased from ATCC. The HEK293T and NIH3T3 cells were cultured in DMEM supplemented with 10% FBS and 1% penicillin/streptomycin. The Jurkat cells were cultured in RPMI1640 supplemented with 10% FBS, 1% penicillin/streptomycin and 1% HEPES, pyruvate, β-ME. All reagents used for cell culture were obtained from Thermo Fisher Scientific. The conditionally immortalized mouse podocyte cell line (MPC5) was kindly provided by Dr. Youhua Liu (Nanfang Hospital, China). To propagate podocytes, MPC5 cells were cultured at 33 °C in RPMI complete medium in the presence of 10 U/mL of mouse IFN-γ (R&D Systems, Minneapolis, MN). Cell differentiation was induced after transferring the cells to 37 °C in RPMI complete medium in the absence of IFN-γ. Experiments were performed using cells from the passages 5 to 18.

*Cloning PLA2R and its mutants*. The CDS of human PLA2R (NM_007366.5) were synthesized and cloned into pcDNA 3.1 (GenScript Biotech). Mutants for PLA2R were generated by PCR and cloned into pUltra-lentiviral vector by GenBuilder (L00701-50, GenScript Biotech) and verified by sequencing. PLA2R-OVA was created by adding the OVA_323-339_ between the N-terminal CFC region (CysR, FNII, CTLD1) and the CTLD2 structural domain of PLA2R. Primers sequences are shown in Table S1, and the amino acid sequences are shown in Table S2.

*Lentivirus production and titration.* Lentiviral vectors were purified using NucleoBond Plasmid kit (Macherey-Nagel). For lentivirus production, 293T cells were seeded in 10 cm cell culture dishes. Virus packaging was performed using TransIT-293 Transfection Reagent (Mirus). Supernatant was collected at 48 and 72 h after transfection, filtered with 0.45 µm filter (Millipore), and subsequently concentrated by ultracentrifugation at 25,000 rpm for 120 min. The virus pellet was dissolved in PBS, aliquoted and stored at -80 °C. Lentivirus stock was titrated on Jurkat cells using GFP as a reporter, as previously reported^76^. The concentration of lentivirus was calculated as follows: infection unit/mL = percent of infection × cell number × 1,000/X μL (where X is the volume of virus added).

*Generation of stable cell lines expressing human PLA2R*. Cells were infected with PLA2R or mutant-expressing lentivirus and used flow cytometry to sort the GFP-positive cells (lentivirus-infected) for generating stable-expressing cell lines. After sorting, cells were passaged 5 times before conducting the experiment.

*Recombinant protein expression and purification*. The extracellular domain of human PLA2R (NM_007366.5) or human THSD7A (NM_015204.3) fused with a 6 x his-tag was cloned into a pcDNA3.1 vector. The constructs were transfected into HEK293 cells with Lipofectamine Reagent (Thermo Fisher Scientific). Supernatant was collected 48 h post transfection and subjected to purification with affinity chromatography. To ensure PLA2R protein structural stability, the purified PLA2R protein was aliquoted and stored at -80 °C, and freeze-thaw cycles were limited to only once.

*Generation of fluorophore-conjugated protein.* PLA2R protein were labeled with EZ-Link™ NHS-LC-Biotin (21336, Thermo Scientific) following the manufacturer's protocol. Briefly, PLA2R protein was incubated with a 20-fold molar excess of NHS-LC-Biotin dissolved in DMSO for 30mins at room temperature. The reaction was quenched by adding 1 M Tris-HCl (pH 7.4) and incubating for an additional 30 min. To remove excess biotin, the reaction mixture was loaded into an Amicon Ultra-0.5 mL 10 kDa centrifugal filter (UFC501096, Millipore) and centrifuged at 14,000 g for 10 min. The labeled protein was washed three times with 1 × PBS. Finally, the concentrated biotinylated protein was collected by inverting the filter into a fresh collection tube and centrifuging at 1,000 g for 2 min. The biotinylated PLA2R was mixed with PE Streptavidin (554061, BD Biosciences) or APC-streptavidin (554067, BD Biosciences) through four sequential additions with a final 4:1 molar ratio of PE-streptavidin or APC-streptavidin to biotinylated PLA2R at 15 min intervals. The fluorescently conjugated protein was stored at 4 °C in the dark for short-term use or at -80 °C for long-term preservation.

*Recombinant antibody production and verification*. Recombinant antibodies were generated as previously described 45. In brief, fresh peripheral blood mononuclear cells (PBMCs) were collected from PMN patients who had not received immune-suppressive therapies. CD19^+^ B cells were enriched using magnetic beads, followed by staining with anti-CD20, anti-CD27 and anti-human IgG. Human Fc block was applied to minimize non-specific staining. PE-conjugated and APC-conjugated PLA2R were utilized to label PLA2R-specific memory B cells. Single cells exhibiting double positivity for PLA2R-PE and PLA2R-APC were sorted into 96-well plates containing 10 µL 10 mM Tris (pH 8.0) with 1u/μL of RNasin Ribonuclease Inhibitor (N2515, Promega). The heavy and light chains of the B cell receptors were amplified using nested PCR. The variable regions of these heavy and light chains were then sequenced and cloned into a pcDNA3.1 expression vector, in-frame fused to the Fc region of IgG1 or IgG4. For antibody production, paired heavy and light chain plasmids were co-transfected into HEK293 cells. The resulting antibodies, designated Ab1-IgG1, Ab3-IgG1 and Ab3-IgG4, were purified using Protein A/G affinity chromatography. The integrity and properties of the antibodies were determined by western blot and ELISA.

*Flow cytometry*. For surface staining, the supernatant was removed by centrifugation and cells were washed with FACS Buffer (PBS with 0.5% BSA). Cells were washed with FACS buffer and stained with fluorochrome-conjugated antibodies on ice for 30 min. After wash, cells were analyzed with FACSCanto II (BD Biosciences).

*Western blot*. Cells were lysed in SDS loading buffer supplemented with 1% β-ME and boiled. After cooling, samples were resolved run on 6%-8% SDS-PAGE gels and transferred to nitrocellulose membrane (Bio-Rad). Membranes were blocked with 5% skim milk in buffer for 1 h at room temperature, followed by overnight incubation with primary antibodies at 4 °C. After washing with TBS containing 0.5% Tween-20, membranes were incubated with HRP-conjugated secondary antibodies (Jackson ImmunoResearch Laboratories) for 1 h at room temperature. Signals were detected using ECL (Thermo Fisher Scientific).

*Immunoprecipitation*. For surface proteins, cells were first incubated with serum and then lysed with IP lysis buffer (PR20037, Proteintech). Cell lysates were centrifuged at 10,000 g for 5 min, and the supernatants were harvested. Protein A/G agarose beads (sc-2003, Santa Cruz) were added to the supernatants and incubated overnight at 4 °C with rotation. After two washs, loading buffer was added to the beads to elute the bound proteins, which were subsequently analyzed by western blot. For biotinylated protein precipitation, cells were incubated with Sulfo-NHS-SS-biotin (final concentration of 0.3 mg/mL) on ice for 1 h. Cells were then lysed in IP lysis buffer and centrifuged at 10,000 g for 5 min, after which the supernatants were collected and incubated with Streptavidin Magnetic Beads at 4 °C overnight with rotation. Beads were washed with PBS the following day, and bound proteins were eluted with loading buffer. For proteins in the supernatant, conditioned medium was centrifuged at 10,000 g for 5 min to removed cell debris. The clarified supernatant was incubated with Protein A/G agarose beads overnight at 4 °C with rotation. Beads were washed with PBS the next day, and bound proteins were eluted with loading buffer.

*Deglycosylation*. Deglycosylation was performed according to the manufacturer's instruction (P0705S, New England Biolabs). Briefly, cell lysate was mixed with Glycoprotein Denaturing Buffer, followed by heating at 100 °C for 10 min. The GlycoBuffer 2, NP40 and PNGase F were then added after chilling the denatured glycoprotein on ice, and incubated at 37 °C for 1 h. The deglycosylated samples were evaluated by western blot.

*RT-PCR (Real-time PCR)*. Total RNA was isolated using TRIzol reagent (T9424, Sigma-Aldrich) and reverse transcribed into cDNA using HiScript SuperMix (Vazyme). Primers were designed using the OligoAnalyzer tool from Integrated DNA Technologies (IDT). Primer sequence is listed in Table S1.

*Immunofluorescence*. Cells were fixed with 4% PFA for 15 min. After washing with PBS, cells were permeabilized with PBST (0.3% Triton X-100 in PBS) for 15 min. Cells were blocked with 10% normal donkey serum for 30 min, followed by primary antibody staining for 2 h at room temperature. Cells were then washed 3 times and incubated with fluorochrome-labeled secondary antibody for 1 h at room temperature. Images were taken by confocal microscopy (FV1200, Olympus).

*Immunohistochemistry staining.* Formalin-fixed, paraffin-embedded tissue underwent heat-induced epitope retrieval in 10 mM citrate buffer (pH 6.0) at 95 °C for 20 min. Endogenous peroxidase activity was quenched with 3% H2O2 in methanol for 15 min. Non-specific binding was blocked with 5% normal goat serum (Vector Laboratories) for 30 min at room temperature. Sections were incubated with primary antibodies at optimized concentrations overnight at 4 °C. After PBS washes (3 x 5 min), sections were incubated with HRP-conjugated secondary antibodies for 30 min at 37 °C. Antigen-antibody complexes were visualized using 3,3'-diaminobenzidine (DAB) chromogen for 5 min. Nuclei were counterstained with Harris hematoxylin for 45 s. Sections were dehydrated, cleared in xylene and mounted. Stained sections were visualized under a light microscope (Olympus).

*Isolation of human lymph node stromal cells for flow cytometry.* Fresh human lymph node tissue was digested in RPMI-based medium containing 2.4 mg/mL Dispase II, 0.6 mg/mL Collagenase P, and 0.3 mg/mL DNase I at 37 °C. Sequential 10 min digestions were performed, and supernatants were collected at each interval into quenching buffer (2% FBS, 5 mM EDTA in 1 × PBS). Pooled cells were centrifuged and processed for CD45 depletion. For CD45 depletion, cells were incubated with CD45-biotin antibody for 5 min at room temperature, washed in MACS buffer (0.5% BSA, 2 mM EDTA in 1 × PBS), and resuspended at 10⁷ cells per 80 μL buffer. Anti-biotin microbeads (Miltenyi Biotec) were then added at 20 μL per 10⁷ cells and incubated for 15 min on ice. CD45^+^ cells were removed using magnetic column separation (Miltenyi Biotec). CD45-depleted cells were blocked with human Fc blocking antibody for 5 min and subsequently stained with antibodies in the presence of viability dye. After fixation and permeabilization (BD Transcription Factor Buffer Set), intracellular staining for PLA2R-AF647 was performed. Data acquisition was carried out on a BD FACSymphony™ A1 cytometer, and data were analyzed using FlowJo v10.8 software.

*BMDC (Bone marrow-derived dendritic cells) culture and lentiviral-transduction*. BM cells were flushed from femurs and tibiae of 6 to 8 weeks old C57BL/6j male mice and transferred into a 15 mL tube. After removing red blood cells with erylysis buffer, cells were counted and seeded at a density of 2 x 10^6^ per well of 24-well plate in RPMI1640 media containing 10% FCS and 20 ng/mL mouse GM-CSF (PeproTech). Half of the medium was removed every 3 days and new medium supplemented with GM-CSF (40 ng/ml) was added. BMDs were used on day 7. For lentivirus transduction, BM cells were mixed with lentivirus at multiplicity of infection (MOI) = 10 and spin-infected at 800 g for 2 h. Lentivirus infection was repeated on day 4. BMDCs were used for the following experiment on day 7 after culture.

*Antigen presentation assay*. Spleen and lymph nodes were collected from 8 to 12 weeks old OTII male mice (Jackson Laboratories) and used for CD4^+^ T cell isolation. Naive CD4^+^ T cells were isolated using MojoSort Mouse CD4 Naïve T Cell Isolation Kit (480040, Biolegend) according to the manufacturer instruction. 1 x 10^5^ CD4^+^ T cells labeled with 5 μM CFSE (Thermo Fisher Scientific) were cocultured with 2 x 10^4^ BMDC-transduced with empty-lentivirus or PLA2R-OVA-expressing lentivirus in U-bottom 96-well plate for 3 days. CFSE dilution and expression of CD25 and CD69 were determined by flow cytometry. Supernatant was harvested for detection of IFN-γ using ELISA kit (Biolegend).

*Adoptive T cell transfer.* Naive CD4^+^ T cells were isolated from CD45.1^+^ OT-II mice using MojoSort Mouse CD4 Naive T Cell Isolation Kit (Biolegend). A total of 2 × 10⁶ naive CD4⁺ T cells were adoptively transferred into CD45.2⁺ recipient mice via retro-orbital injection. Twenty-four hours later, mice were given an intraperitoneal injection of 1 μg LPS per 25 g mouse (InvivoGen). Thirty minutes after LPS administration, 1 × 10⁶ PLA2R-OVA or PLA2R-TR11-OVA-expressing BMDCs pretreated with the indicated sera were subcutaneously injected into the right flank. On day 6 post-BMDC transfer, mice were euthanized, and inguinal lymph nodes were collected for flow cytometric analysis.

*Statistical Analysis.* Statistical analyses were performed using PRISM software (GraphPad). Data are presented as mean ± SEM and analyzed by Student's t-test and one-way ANOVA followed by Tukey multiple-comparison test. A *P*-value less than 0.05 was considered statistically significant.

## Supplementary Material

Supplementary figures and tables.
