# Supplementary material for: Antibody-induced internalization and degradation of PLA2R amplifies CD4+ T cell activation
Source: Theranostics. 2026 Jan 1;16(1):37–57. doi: 10.7150/thno.123035 (PMC12665110; doi:10.7150/thno.123035)
Supplement: Supplementary file 1 — Supplementary figures and tables. [file thnov16p0037s1.pdf]

Supplementary Figures

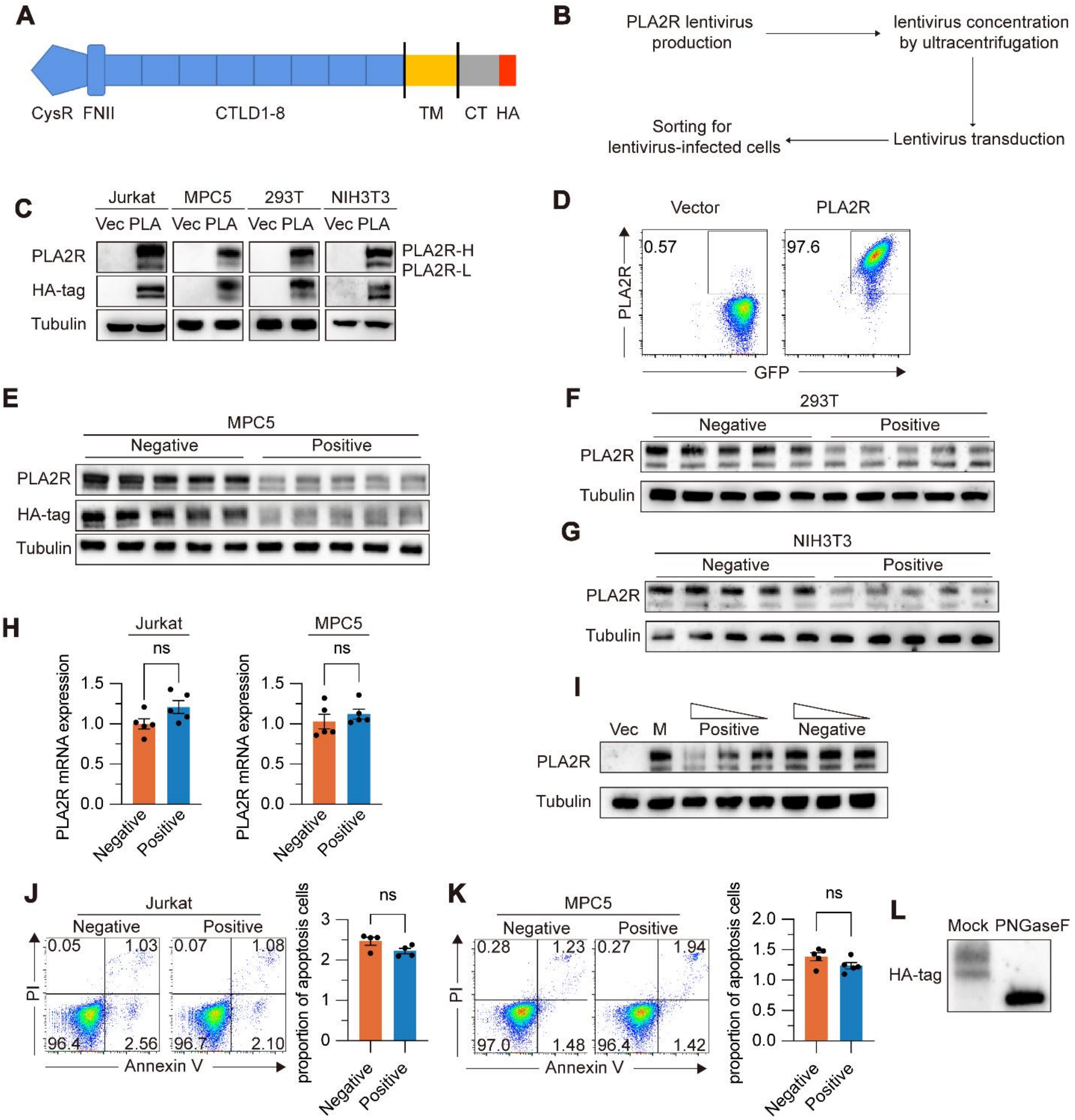

Figure S1. Lentivirus-mediated PLA2R overexpression and verification. (**A**) Schematic diagram of PLA2R-overexpression construct. HA-tag is added after the C-terminus of PLA2R. (**B**) Process for generating lentivirus-infected stable cell line. (**C**) Validation of PLA2R overexpression in multiple PLA2R stable expressing cell lines. PLA2R was detected with anti-PLA2R or HA-tag antibodies. Tubulin was used as a loading control. (**D**) Flow cytometry detection of PLA2R on the surface of PLA2R-Jurkat cells. Experiments were repeated three times with similar results. (**E-G**) Western blot analysis of PLA2R expression in PLA2R-MPC5

cells in **E**, PLA2R-293T cells in **F**, or PLA2R-NIH3T3 cells in **G,** treated with Negative (n = 5) or Positive (n = 5) serum. PLA2R expression was detected with anti-PLA2R (**E**-**G**) or anti-HA-tag (**E**). (**H**) Real-time PCR analysis of PLA2R expression in PLA2R-Jurkat or PLA2R-MPC5 cells treated with Negative (n = 5) or Positive (n = 5) serum. (**I**) Western blot analysis of PLA2R expression in PLA2R-Jurkat cells treated with serial dilutions of Positive serum (5, 1, and 0.5 RU/mL, from high to low) or an equal volume of Negative serum as control. (**J**-**K**) PLA2R-expressing-Jurkat (**J**) or -MPC5 (**K**) cells were treated with Negative (n = 4 for Jurkat cells and n = 5 for MPC5 cells) or Positive serum (n = 4 for Jurkat cells and n = 5 for MPC5 cells) for 6 h and subjected to flow cytometry analysis of PI and annexin V staining. PI, propidium iodide. (**L**) Western blot detection of PLA2R expression without (Mock) or with PNGaseF treatment. Data are shown as the mean ± SEM. Statistical significance was assessed using 2-tailed, unpaired Student's t test. ns, non-significant. Experiments were repeated twice with similar results.

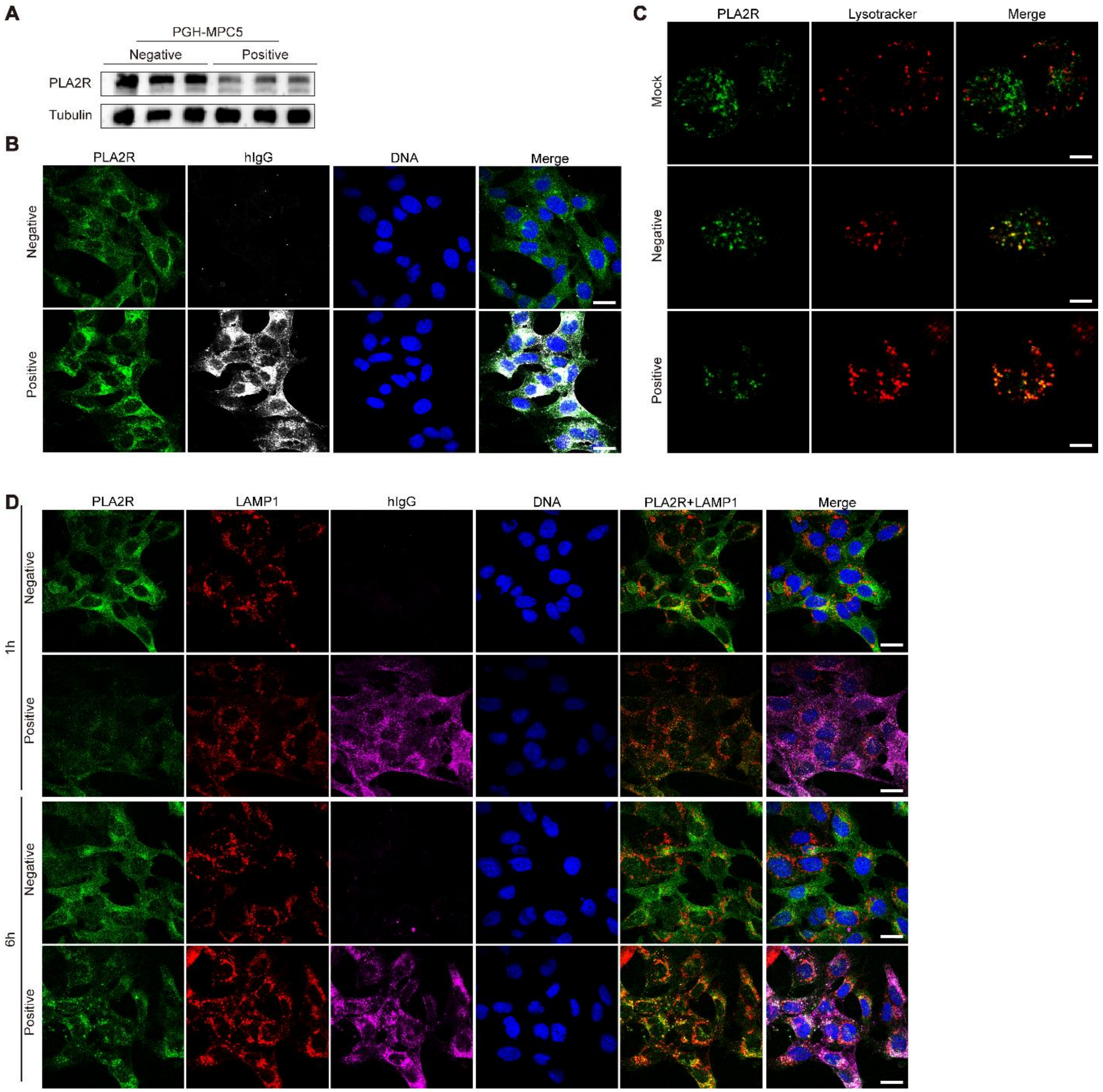

Figure S2. HIgG enters the cell and colocalizes with PLA2R. (**A**) Western blot analysis of PLA2R expression in PGH-MPC5 cells treated for 6 h with Negative (n = 3) or Positive (n = 3) serum. (**B**) Confocal analysis of PLA2R and hIgG in PGH-MPC5 cells treated for 6 h with Negative or Positive serum. Scale bar = 20 µm. (**C**) PGH-MPC5 cells were incubated without (Mock), or with Negative or Positive serum. Lysosomes were stained with lysotracker. The spatial relationship between PLA2R-GFP (green) and lysotracker (red) was analyzed by confocal microscopy. Scale bar: 5 µm. (**D**) Confocal analysis of PLA2R, LAMP1 and hIgG in PGH-MPC5 treated for 1 h or 6 h with Negative or Positive serum. Scale bar: 20 µm. Experiments were repeated twice with similar results.

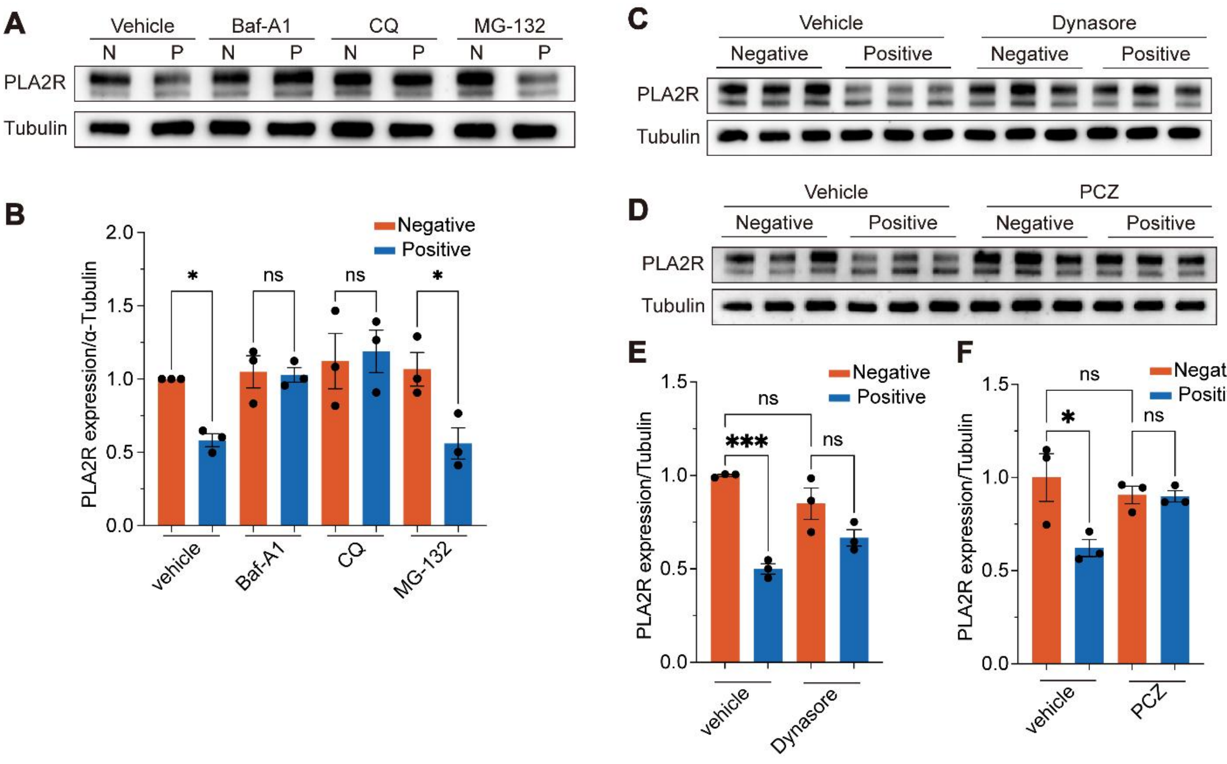

Figure S3. Inhibition reverse PLA2R-Ab$^+$ serum-induced decrease in PLA2R expression. (**A**-**B**) Western blot analysis of PLA2R expression in PLA2R-MPC5 cells treated with Negative (N, n = 3) or Positive (P, n = 3) serum in the presence of lysosomal inhibitors (Baf-A1 and CQ) or proteasome inhibitor (MG-132). Representative and quantification data are shown in **A** and **B**, respectively. Data are presented as the mean ± SEM. Experiments were repeated three times with similar results. Statistical significance was assessed using one-way ANOVA followed by Newman-Keuls multiple-comparison test (B). ns, non-significant, *$P < 0.05$. (**C**-**F**) Western blot analysis of PLA2R expression in PLA2R-Jurkat cells treated with Negative (n = 3) or Positive serum (n = 3) in the presence of indicated inhibitors. Representative and quantification data are shown in **C** and **E** for Dynasore, and **D** and **F** for PCZ, respectively. Data are shown as the mean ± SEM (**C** and **D**). Experiments were repeated twice with similar results. Statistical significance was assessed using one-way ANOVA followed by Tukey multiple-comparison test (**C** and **D**). ns, non-significant, *$P < 0.05$, ***$P < 0.001$.

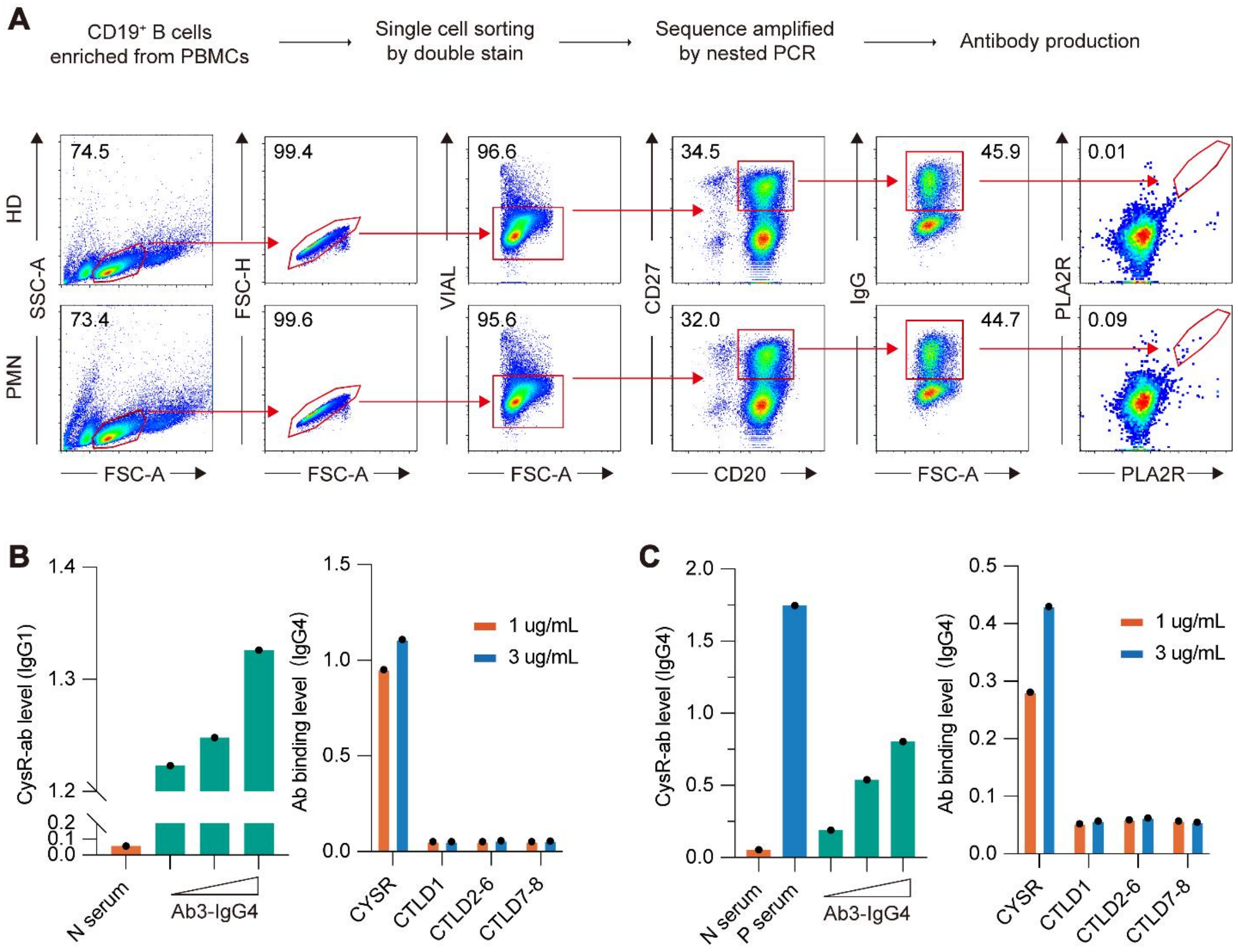

Figure S4. Ab3 specifically recognizes the CysR domain of PLA2R. (**A**) Schematic diaphragm for recombinant antibody production and gating strategy for sorting of PLA2R-specific B-cells. (**B** and **C**) ELISA analysis of the reactivity of Ab3-IgG1 (0.3/1/3 μg/mL) in **B** and Ab3-IgG4 (0.1/1/10 μg/mL) in **C** to the CysR domain and other domains (including CTLD1, CTLD2-6 and CTLD7-8) of PLA2R. HD, Healthy donor; PMN, primary membranous nephropathy patient; LOD, limit of detection. Experiments were repeated three times with similar results.

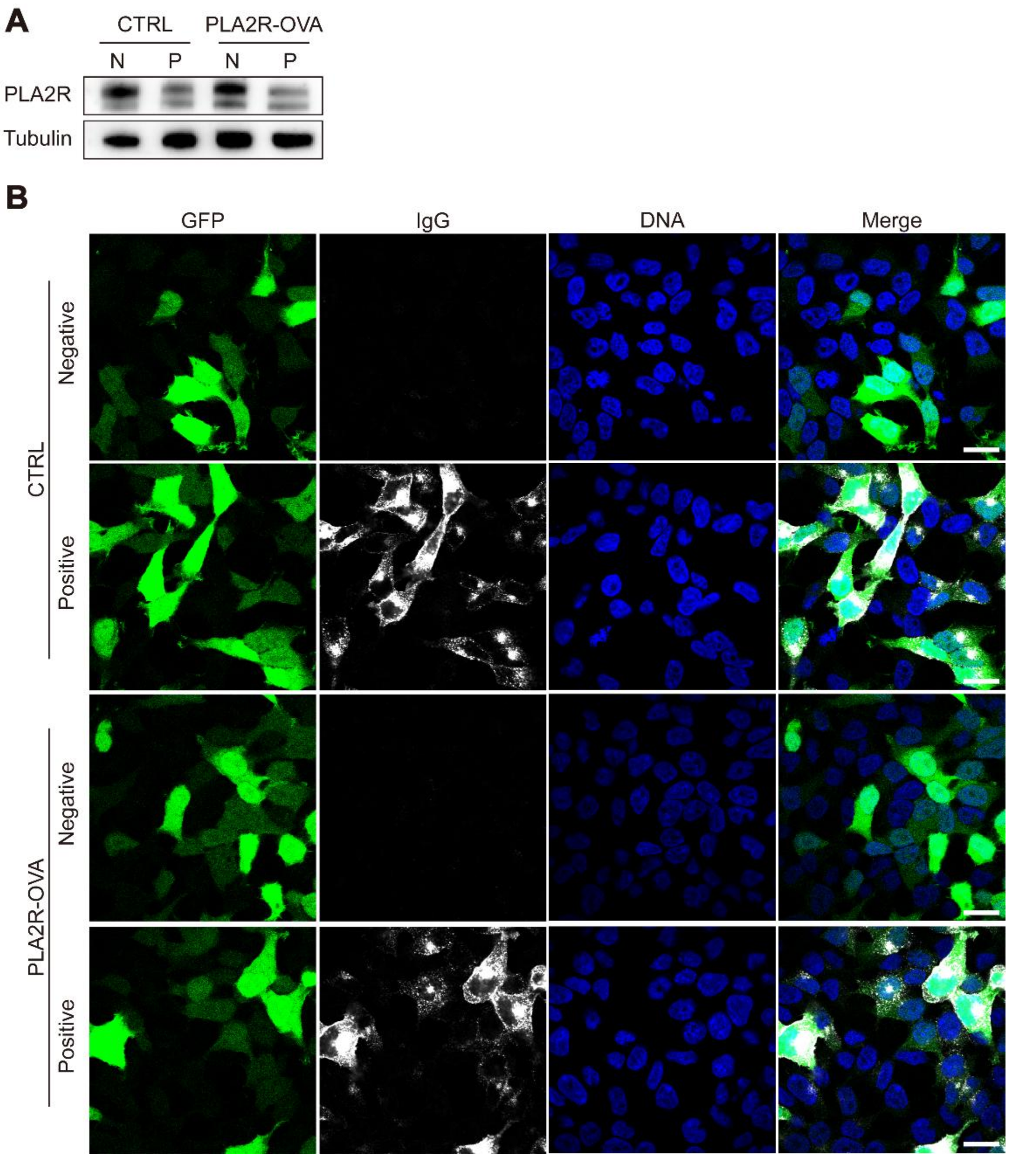

Figure S5. PLA2R-Ab⁺ serum induces endocytosis and degradation of PLA2R-OVA. (**A**) Western blot analysis of PLA2R expression in PLA2R-293T or PLA2R-OVA-293T cells treated with Negative or Positive serum. (**B**) Subcellular localization of human IgG (white) was evaluated by confocal microscopy after fixation. GFP-positive cells are lentivirus-infected cells. Scale bar: 20 μm. Experiments were repeated twice with similar results.

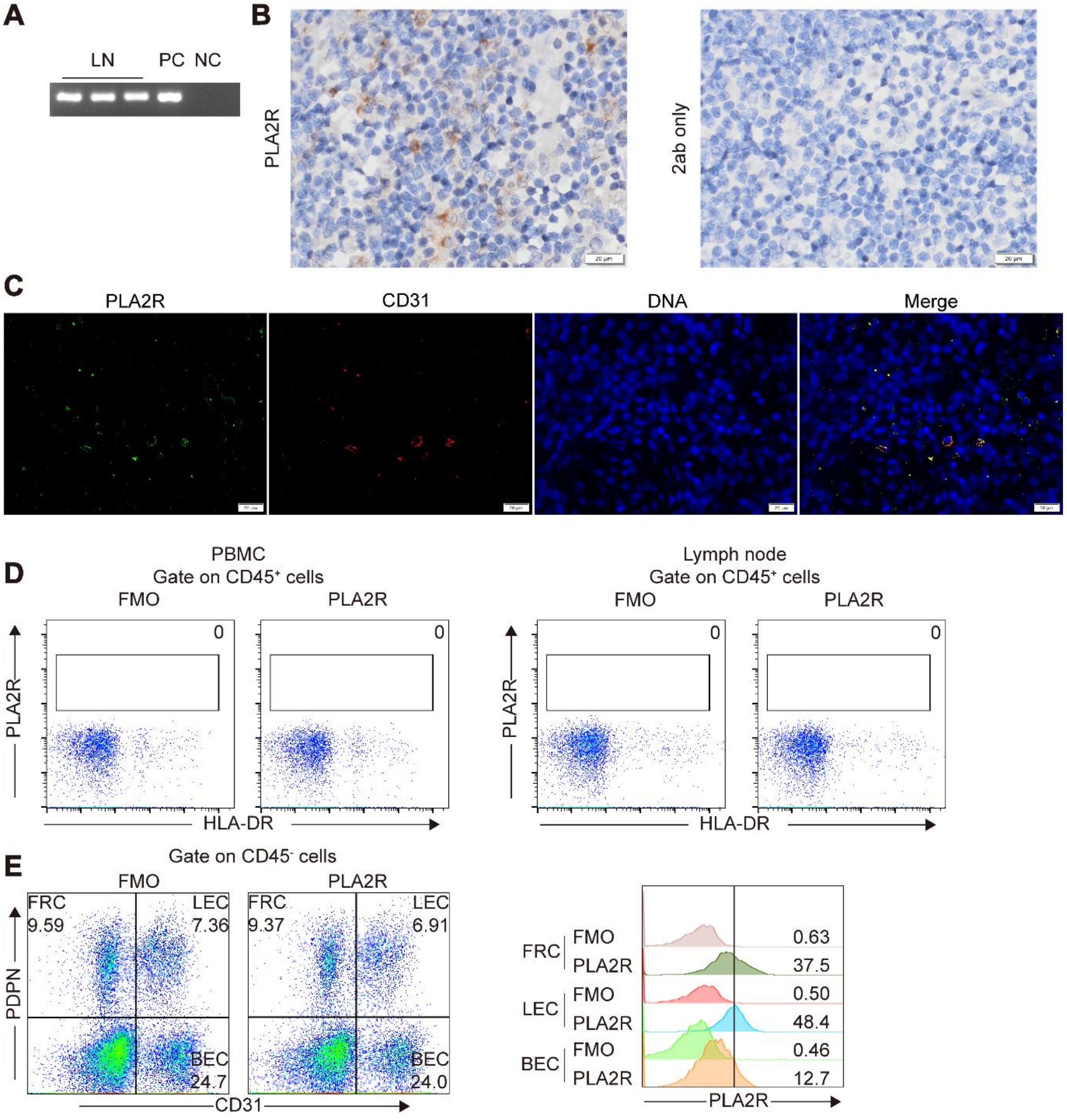

Figure S6. PLA2R expression in human lymph node. (**A**) mRNA expression of PLA2R in lymph node (LN). PLA2R mRNA was amplified by PCR and visualized via agarose gel electrophoresis. Positive control (PC), Jurkat-PLA2R; Negative control (NC), Jurkat cells. (**B**) IHC staining for PLA2R protein expression in lymph node. Secondary antibody only (2ab only) was used as negative control for staining. (**C**) Immunofluorescence for PLA2R with CD31 (endothelial marker) in lymph node. Scale bar: 20 μm. (**D**) Flow cytometry analysis of PLA2R expression in CD45-positive ($CD45^+$) cells of PBMC and lymph node cells. (**E**) Flow cytometry analysis of PLA2R expression in isolated lymph node stromal cells. PBMC, Peripheral blood mononuclear cells; PDPN, Podoplanin; FRC, Fibroblastic reticular cells; LEC, lymphatic endothelial cells; BEC, blood endothelial cells; FMO, fluorescence minus one.

Supplementary Tables

Table S1. Primer sequences used in this study.

Primer		sequences
Human PLA2R	Forward	AAGAGGGATGGGAGAGACA
	Reverse	GGTTACAAGTGCAGGAGGA
Human beta-actin	Forward	AGCGTGCCTTTGTTCACT
	Reverse	CTGCTCCAACCTCCTCATAATC
Y19A fragment A	Forward	GAGGAGAATCCCGGGCCTTCTAGAATGCTGCTG
	Reverse	TAAAGTTGGTTGCAGGAGCGTAAGGATTCCGAA
Y19A fragment B	Forward	TTTCGGAATCCTTACGCTCCTGCAACCAACTTTA
	Reverse	GAACGAATTCTGATCACACGAATTCTTAAGCGTAATCTGG
TR11 fragment A	Forward	GAGGAGAATCCCGGGCCTTCTAGAATGCTGCTG
	Reverse	TAAAGTTGGTTGCAGGAGCGTAAGGATTCCGAA
TR11 fragment B	Forward	GAGAATCCCGGGCCTTCTAGAATGCTGCTG
	Reverse	GAATTCTGATCACACGAATTCTTAAGCGTAATCTGGAACATCGTAT GGGTAAAGTCTCCTGAAGAAGCCA
PGH fragment A	Forward	CTCACTATAGGGAGACCCAAGCTGGCTAGCATGCTGCTGTCGCCGT CGCT
	Reverse	CCCTTGCTGAGCTCGGTACCTTGGTCACTCTTCTCAAGAT
PGH fragment B	Forward	GTGACCAAGGTACCGAGCTCAGCAAGGGCGAGGAGCTGTT
	Reverse	GCCGCCACTGTGCTGGATATCTGCAGAATTCTTAAGCGTAATCTGG AACATCGTATGGGTACTTGTACAGCTCGTCCATGC
PLA2R-OVA fragment A	Forward	GAGGAGAATCCCGGGCCTTCTAGAATGCTGCTG
	Reverse	CCATCTGCCTGCTTCATTGATTTCTGCATGTGCTGCATGGACAGCTT GAGATATGCCAGGCTCACAGTGGGTAGC
PLA2R-OVA	Forward	TGCAGAAATCAATGAAGCAGGCAGATGGAATCCCTACAATCGTAAT

fragment B		TGCT
	Reverse	GAGTCGACGACTCCGGAACGAATTCTTAAGCGTAATCTGG

Table S2. Amino Acid sequences used in this study

Protein	Amino Acid sequences
PLA2R	MLLSPSLLLLLLLGAPRGCAEGVAAALTPERLLEWQDKGIFVIQSESLKKCIQAGKSVLTLENCKQANK<br>WKWVSNHGLFNIGGSGCLGLNFSAPEQPLSLYECDSTLVSLRWRCNRKMITGPLQYSVQVAHDNTVVA<br>YIHKWISYGSGGGDICEYLHKDLHTIKGNTHGMPCMFPFQYNHQWHHECTREGREDDLLWCATTSRY<br>EKWGFCPDPTSAEVGCDTIWEKDLNSHICYQFNLLSSLSWSEAHSSCQMQGGTLLSITDETEENFIREH<br>KTVEVWMGLNQLDEHAGWQWSDGTPLNYLNWSPEVNFEPFVEDHCGTFSSFMPSAWRSRDCESTLP<br>KYLNHIDHEIVEKDAWKYYATHCEPGWNPYNRNCYKLQKEEKTWHEALRSCQADNSALIDITSLAEV<br>TLLGDENASETWIGLSSNKIPVSFEWSNDSSVIFTNWHTLEPHIFPNRSQLCVSAEQSEGHWKVKNCEE<br>ICKKAGHVLSDAESGCQEGWERHGGFCYKIDTVLRSFDQASSGYYCPPALVTITNRFEQAFITSLISSVV<br>DSYFWIALQDQNDTGEYTWKPVGQKPEPVQYTHWNTHQPRYSGGCVAMRGRHPLGRWEVKHCRHF<br>SLCKQPVENQEKAEYEERWPFHPCYLDWESEPGLASCFKVFHSEKVLMKRTWREAEAFCEEFGAHLA<br>IEEENFVNELLHSKFNWTEERQFWIGFNKRNPLNAGSWEWSDRTPVVSSFLDNTYFGEDARNCAVYKA<br>LLPLHCGSKREWICKIPRDVKPKIPFWYQYDVPWLFYQDAEYLFHTFASEWLNFEFVCSWLHSDLLTIH<br>EQEFIHSKIKALSKYGASWWIGLQEERANDEFRWRDGTPVIYQNWDTGRERTVNNQSQRCGFISSITGL<br>EECSVSMPSICKRKKVWLIEKKKDTPKQHGTCPKGWLYFNYKCLLLNIPKDPSSWKNWTHAQHFCAE<br>TLVAIESEVEQAFITMNLFGQTTSVWIGLQNDDYETWLNGKPVVYSNWSPFDIINIPSHNTTEVQKHIPL<br>LSSNPNFHFTGKWYFEDCGKEGYGFVCEKMQDTSGHGVNTSDMYPMPNTLEYGNRTYKIINANMTW<br>KTCLMHKAQLVSITDQYHQSFLTVVLNRLGYAHWIGLFTTDNGLNFDWSDGTKSSFTFWKDEESSLLG<br>FADSNGRWHSTACESFLQGAICHVPPETRQSEHPELCSETSIPWIKFKSNCYSFSTVLDSMSFEAAHEFC<br>GSNLLTIKDEAENAFLLEELFAFGSSVQMVWLNAQFDGNNETIKWFDGTPTDQSNWGIRKPDTDYFKP<br>VALRIPEGLWQLSPCQEKKGFICKMEADIHTAEALPEKGPSHSIIPLAVVLTLIVIVAICTLSFCIYKHNGG<br>LAGFRNPYYPATNFSTVYLEENILISDLEKSDQYPYDVPDYA*
PGH	MLLSPSLLLLLLLGAPRGCAEGVAAALTPERLLEWQDKGIFVIQSESLKKCIQAGKSVLTLENCKQAN<br>KHMLWKWVSNHGLFNIGGSGCLGLNFSAPEQPLSLYECDSTLVSLRWRCNRKMITGPLQYSVQVAH<br>DNTVVASRKYIHKWISYGSGGGDICEYLHKDLHTIKGNTHGMPCMFPFQYNHQWHHECTREGREDD<br>LLWCATTSRYERDEKWGFCPDPTSAEVGCDTIWEKDLNSHICYQFNLLSSLSWSEAHSSCQMQGGTL<br>LSITDETEENFIREHMSSKTVEVWMGLNQLDEHAGWQWSDGTPLNYLNWSPEVNFEPFVEDHCGTF<br>SSFMPSAWRSRDCESTLPYICKKYLNHIDHEIVEKDAWKYYATHCEPGWNPYNRNCYKLQKEEKTW<br>HEALRSCQADNSALIDITSLAEVEFLVTLLGDENASETWIGLSSNKIPVSFEWSNDSSVIFTNWHTLEP<br>HIFPNRSQLCVSAEQSEGHWKVKNCEERLFYICKKAGHVLSDAESGCQEGWERHGGFCYKIDTVLRS

FDQASSGYYCPPALVTITNRFEQAFITSLISSVVKMKDSYFWIALQDQNDTGEYTWKPVGQKPEPVQY
THWNTHQPRYSGGCVAMRGRHPLGRWEVKHCRHFKAMSLCKQPVENQEKAEYEERWPFHPCYLD
WESEPGLASCFKVFHSEKVLMKRTWREAEAFCEEFGAHLASFAHIEEENFVNELLHSKFNWTEERQF
WIGFNKRNPLNAGSWEWSDRTPVVSSFLDNTYFGEDARNCAVYKANKTLLPLHCGSKREWICKIPRD
VKPKIPFWYQYDVPWLFYQDAEYLFHTFASEWLNFEFVCSWLHSDLLTIHSAHEQEFIHSKIKALSKY
GASWWIGLQEERANDEFRWRDGTPVIYQNWDTGRERTVNNQSQRCGFISSITGLWGSEECSVSMPSI
CKRKKVWLIEKKKDTPKQHGTCPKGWLYFNYKCLLLNIPKDPSSWKNWTHAQHFCAEEGGTLVAIE
SEVEQAFITMNLFGQTTSVWIGLQNDDYETWLNGKPVVYSNWSPFDIINIPSHNTTEVQKHIPLCALL
SSNPNFHFTGKWYFEDCGKEGYGFVCEKMQDTSGHGVNTSDMYPMPNTLEYGNRTYKIINANMTW
YAAIKTCLMHKAQLVSITDQYHQSFLTVVLNRLGYAHWIGLFTTDNGLNFDWSDGTKSSFTFWKDEE
SSLLGDCVFADSNGRWHSTACESFLQGAICHVPPETRQSEHPELCSETSIPWIKFKSNCYSFSTVLDSM
SFEAAHEFCKKEGSNLLTIKDEAENAFLLEELFAFGSSVQMVWLNAQFDGNNETIKWFDGTPTDQSN
WGIRKPDTDYFKPHHCVALRIPEGLWQLSPCQEKKGFICKMEADIHTAEALPEKGPSHSIIPLAVVLTLI
VIVAICTLSFCIYKHNGGFFRRLAGFRNPYYPATNFSTVYLEENILISDLEKSDQGTELSKGEELFTGVV
PILVELDGDVNGHKFSVSGEGEGDATYGKLTLKFICTTGKLPVPWPTLVTTLTYGVQCFSRYPDHMKQ
HDFFKSAMPEGYVQERTIFFKDDGNYKTRAEVKFEGDTLVNRIELKGIDFKEDGNILGHKLEYNYNS
HNVYIMADKQKNGIKVNFKIRHNIEDGSVQLADHYQQNTPIGDGPVLLPDNHYLSTQSALSKDPNEK
RDHMVLLEFVTAAGITHGMDELYKYPYDVPDYA*

MLLSPSLLLLLLLGAPRGCAEGVAAALTPERLLEWQDKGIFVIQSESLKKCIQAGKSVLTLENCKQAN
KHMLWKWVSNHGLFNIGGSGCLGLNFSAPEQPLSLYECDSTLVSLRWRCNRKMITGPLQYSVQVAH
DNTVVASRKYIHKWISYGSGGGDICEYLHKDLHTIKGNTHGMPCMFPFQYNHQWHHECTREGREDD
LLWCATTSRYERDEKWGFCPDPTSAEVGCDTIWEKDLNSHICYQFNLLSSLSWSEAHSSCQMQGGTL
LSITDETEENFIREHMSSKTVEVWMGLNQLDEHAGWQWSDGTPLNYLNWSPEVNFEPFVEDHCGTF
SSFMPSAWRSRDCESTLPYICKKYLNHIDHEIVEKDAWKYYATHCEPGWNPYNRNCYKLQKEEKTW
HEALRSCQADNSALIDITSLAEVEFLVTLLGDENASETWIGLSSNKIPVSFEWSNDSSVIFTNWHTLEP
HIFPNRSQLCVSAEQSEGHWKVKNCEERLFYICKKAGHVLSDAESGCQEGWERHGGFCYKIDTVLRS
FDQASSGYYCPPALVTITNRFEQAFITSLISSVVKMKDSYFWIALQDQNDTGEYTWKPVGQKPEPVQY
TR11 THWNTHQPRYSGGCVAMRGRHPLGRWEVKHCRHFKAMSLCKQPVENQEKAEYEERWPFHPCYLD
WESEPGLASCFKVFHSEKVLMKRTWREAEAFCEEFGAHLASFAHIEEENFVNELLHSKFNWTEERQF
WIGFNKRNPLNAGSWEWSDRTPVVSSFLDNTYFGEDARNCAVYKANKTLLPLHCGSKREWICKIPRD
VKPKIPFWYQYDVPWLFYQDAEYLFHTFASEWLNFEFVCSWLHSDLLTIHSAHEQEFIHSKIKALSKY
GASWWIGLQEERANDEFRWRDGTPVIYQNWDTGRERTVNNQSQRCGFISSITGLWGSEECSVSMPSI
CKRKKVWLIEKKKDTPKQHGTCPKGWLYFNYKCLLLNIPKDPSSWKNWTHAQHFCAEEGGTLVAIE
SEVEQAFITMNLFGQTTSVWIGLQNDDYETWLNGKPVVYSNWSPFDIINIPSHNTTEVQKHIPLCALL
SSNPNFHFTGKWYFEDCGKEGYGFVCEKMQDTSGHGVNTSDMYPMPNTLEYGNRTYKIINANMTW
YAAIKTCLMHKAQLVSITDQYHQSFLTVVLNRLGYAHWIGLFTTDNGLNFDWSDGTKSSFTFWKDEE
SSLLGDCVFADSNGRWHSTACESFLQGAICHVPPETRQSEHPELCSETSIPWIKFKSNCYSFSTVLDSM

	SFEAAHEFCKKEGSNLLTIKDEAENAFLLEELFAFGSSVQMVWLNAQFDGNNETIKWFDGTPTDQSN
	WGIRKPDTDYFKPHHCVALRIPEGLWQLSPCQEKKGFICKMEADIHTAEALPEKGPSHSIIPLAVVLTLI
	VIVAICTLSFCIYKHNGGFFRRLYPYDVPDYA*
	MLLSPSLLLLLLLGAPRGCAEGVAAALTPERLLEWQDKGIFVIQSESLKKCIQAGKSVLTLENCKQAN
	KHMLWKWVSNHGLFNIGGSGCLGLNFSAPEQPLSLYECDSTLVSLRWRCNRKMITGPLQYSVQVAH
	DNTVVASRKYIHKWISYGSGGGDICEYLHKDLHTIKGNTHGMPCMFPFQYNHQWHHECTREGREDD
	LLWCATTSRYERDEKWGFCPDPTSAEVGCDTIWEKDLNSHICYQFNLLSSLSWSEAHSSCQMQGGTL
	LSITDETEENFIREHMSSKTVEVWMGLNQLDEHAGWQWSDGTPLNYLNWSPEVNFEPFVEDHCGTF
	SSFMPSAWRSRDCESTLPYICKKYLNHIDHEIVEKDAWKYYATHCEPGWNPYNRNCYKLQKEEKTW
	HEALRSCQADNSALIDITSLAEVEFLVTLLGDENASETWIGLSSNKIPVSFEWSNDSSVIFTNWHTLEP
	HIFPNRSQLCVSAEQSEGHWKVKNCEERLFYICKKAGHVLSDAESGCQEGWERHGGFCYKIDTVLRS
	FDQASSGYYCPPALVTITNRFEQAFITSLISSVVKMKDSYFWIALQDQNDTGEYTWKPVGQKPEPVQY
	THWNTHQPRYSGGCVAMRGRHPLGRWEVKHCRHFKAMSLCKQPVENQEKAEYEERWPFHPCYLD
Y19A	WESEPGLASCFKVFHSEKVLMKRTWREAEAFCEEFGAHLASFAHIEEENFVNELLHSKFNWTEERQF
	WIGFNKRNPLNAGSWEWSDRTPVVSSFLDNTYFGEDARNCAVYKANKTLLPLHCGSKREWICKIPRD
	VKPKIPFWYQYDVPWLFYQDAEYLFHTFASEWLNFEFVCSWLHSDLLTIHSAHEQEFIHSKIKALSKY
	GASWWIGLQEERANDEFRWRDGTPVIYQNWDTGRERTVNNQSQRCGFISSITGLWGSEECSVSMPSI
	CKRKKVWLIEKKKDTPKQHGTCPKGWLYFNYKCLLLNIPKDPSSWKNWTHAQHFCAEEGGTLVAIE
	SEVEQAFITMNLFGQTTSVWIGLQNDDYETWLNGKPVVYSNWSPFDIINIPSHNTTEVQKHIPLCALL
	SSNPNFHFTGKWYFEDCGKEGYGFVCEKMQDTSGHGVNTSDMYPMPNTLEYGNRTYKIINANMTW
	YAAIKTCLMHKAQLVSITDQYHQSFLTVVLNRLGYAHWIGLFTTDNGLNFDWSDGTKSSFTFWKDEE
	SSLLGDCVFADSNGRWHSTACESFLQGAICHVPPETRQSEHPELCSETSIPWIKFKSNCYSFSTVLDSM
	SFEAAHEFCKKEGSNLLTIKDEAENAFLLEELFAFGSSVQMVWLNAQFDGNNETIKWFDGTPTDQSN
	WGIRKPDTDYFKPHHCVALRIPEGLWQLSPCQEKKGFICKMEADIHTAEALPEKGPSHSIIPLAVVLTLI
	VIVAICTLSFCIYKHNGGFFRRLAGFRNPYAPATNFSTVYLEENILISDLEKSDQYPYDVPDYA*
	MLLSPSLLLLLLLGAPRGCAEGVAAALTPERLLEWQDKGIFVIQSESLKKCIQAGKSVLTLENCKQAN
	KHMLWKWVSNHGLFNIGGSGCLGLNFSAPEQPLSLYECDSTLVSLRWRCNRKMITGPLQYSVQVAH
	DNTVVASRKYIHKWISYGSGGGDICEYLHKDLHTIKGNTHGMPCMFPFQYNHQWHHECTREGREDD
	LLWCATTSRYERDEKWGFCPDPTSAEVGCDTIWEKDLNSHICYQFNLLSSLSWSEAHSSCQMQGGTL
	LSITDETEENFIREHMSSKTVEVWMGLNQLDEHAGWQWSDGTPLNYLNWSPEVNFEPFVEDHCGTF
PLA2R-	SSFMPSAWRSRDCESTLPYICKKYLNHIDHEIVEKDAWKYYATHCEPGISQAVHAAHAEINEAGRWNP
OVA	YNRNCYKLQKEEKTWHEALRSCQADNSALIDITSLAEVEFLVTLLGDENASETWIGLSSNKIPVSFEW
	SNDSSVIFTNWHTLEPHIFPNRSQLCVSAEQSEGHWKVKNCEERLFYICKKAGHVLSDAESGCQEGW
	ERHGGFCYKIDTVLRSFDQASSGYYCPPALVTITNRFEQAFITSLISSVVKMKDSYFWIALQDQNDTGE
	YTWKPVGQKPEPVQYTHWNTHQPRYSGGCVAMRGRHPLGRWEVKHCRHFKAMSLCKQPVENQEK
	AEYEERWPFHPCYLDWESEPGLASCFKVFHSEKVLMKRTWREAEAFCEEFGAHLASFAHIEEENFVN
	ELLHSKFNWTEERQFWIGFNKRNPLNAGSWEWSDRTPVVSSFLDNTYFGEDARNCAVYKANKTLLP

	LHCGSKREWICKIPRDVKPKIPFWYQYDVPWLFYQDAEYLFHTFASEWLNFEFVCSWLHSDLLTIHSA<br>HEQEFIHSKIKALSKYGASWWIGLQEERANDEFRWRDGTPVIYQNWDTGRERTVNNQSQRCGFISSIT<br>GLWGSEECSVSMPSICKRKKVWLIEKKKDTPKQHGTCPKGWLYFNYKCLLLNIPKDPSSWKNWTHA<br>QHFCAEEGGTLVAIESEVEQAFITMNLFGQTTSVWIGLQNDDYETWLNGKPVVYSNWSPFDIINIPSH<br>NTTEVQKHIPLCALLSSNPNFHFTGKWYFEDCGKEGYGFVCEKMQDTSGHGVNTSDMYPMPNTLEY<br>GNRTYKIINANMTWYAAIKTCLMHKAQLVSITDQYHQSFLTVVLNRLGYAHWIGLFTTDNGLNFDWS<br>DGTKSSFTFWKDEESSLLGDCVFADSNGRWHSTACESFLQGAICHVPPETRQSEHPELCSETSIPWIKF<br>KSNCYSFSTVLDSMSFEAAHEFCKKEGSNLLTIKDEAENAFLLEELFAFGSSVQMVWLNAQFDGNNE<br>TIKWFDGTPTDQSNWGIRKPDTDYFKPHHCVALRIPEGLWQLSPCQEKKGFICKMEADIHTAEALPEK<br>GPSHSIIPLAVVLTLIVIVAICTLSFCIYKHNGGFFRRLAGFRNPYYPATNFSTVYLEENILISDLEKSDQY<br>PYDVPDYA*
TR11-OVA	MLLSPSLLLLLLLGAPRGCAEGVAAALTPERLLEWQDKGIFVIQSESLKKCIQAGKSVLTLENCKQAN<br>KHMLWKWVSNHGLFNIGGSGCLGLNFSAPEQPLSLYECDSTLVSLRWRCNRKMITGPLQYSVQVAH<br>DNTVVASRKYIHKWISYGSGGGDICEYLHKDLHTIKGNTHGMPCMFPFQYNHQWHHECTREGREDD<br>LLWCATTSRYERDEKWGFCPDPTSAEVGCDTIWEKDLNSHICYQFNLLSSLSWSEAHSSCQMQGGTL<br>LSITDETEENFIREHMSSKTVEVWMGLNQLDEHAGWQWSDGTPLNYLNWSPEVNFEPFVEDHCGTF<br>SSFMPSAWRSRDCESTLPYICKKYLNHIDHEIVEKDAWKYYATHCEPGISQAVHAAHAEINEAGRWNP<br>YNRNCYKLQKEEKTWHEALRSCQADNSALIDITSLAEVEFLVTLLGDENASETWIGLSSNKIPVSFEW<br>SNDSSVIFTNWHTLEPHIFPNRSQLCVSAEQSEGHWKVKNCEERLFYICKKAGHVLSDAESGCQEGW<br>ERHGGFCYKIDTVLRSFDQASSGYYCPPALVTITNRFEQAFITSLISSVVKMKDSYFWIALQDQNDTGE<br>YTWKPVGQKPEPVQYTHWNTHQPRYSGGCVAMRGRHPLGRWEVKHCRHFKAMSLCKQPVENQEK<br>AEYEERWPFHPCYLDWESEPGLASCFKVFHSEKVLMKRTWREAEAFCEEFGAHLASFAHIEEENFVN<br>ELLHSKFNWTEERQFWIGFNKRNPLNAGSWEWSDRTPVVSSFLDNTYFGEDARNCAVYKANKTLLP<br>LHCGSKREWICKIPRDVKPKIPFWYQYDVPWLFYQDAEYLFHTFASEWLNFEFVCSWLHSDLLTIHSA<br>HEQEFIHSKIKALSKYGASWWIGLQEERANDEFRWRDGTPVIYQNWDTGRERTVNNQSQRCGFISSIT<br>GLWGSEECSVSMPSICKRKKVWLIEKKKDTPKQHGTCPKGWLYFNYKCLLLNIPKDPSSWKNWTHA<br>QHFCAEEGGTLVAIESEVEQAFITMNLFGQTTSVWIGLQNDDYETWLNGKPVVYSNWSPFDIINIPSH<br>NTTEVQKHIPLCALLSSNPNFHFTGKWYFEDCGKEGYGFVCEKMQDTSGHGVNTSDMYPMPNTLEY<br>GNRTYKIINANMTWYAAIKTCLMHKAQLVSITDQYHQSFLTVVLNRLGYAHWIGLFTTDNGLNFDWS<br>DGTKSSFTFWKDEESSLLGDCVFADSNGRWHSTACESFLQGAICHVPPETRQSEHPELCSETSIPWIKF<br>KSNCYSFSTVLDSMSFEAAHEFCKKEGSNLLTIKDEAENAFLLEELFAFGSSVQMVWLNAQFDGNNE<br>TIKWFDGTPTDQSNWGIRKPDTDYFKPHHCVALRIPEGLWQLSPCQEKKGFICKMEADIHTAEALPEK<br>GPSHSIIPLAVVLTLIVIVAICTLSFCIYKHNGGFFRRLYPYDVPDYA*

Table S2. Amino Acid sequences used in this study. In the table, red segments designate the HA-tag amino acid sequence, green portions correspond to the GFP amino acid sequence, blue regions indicate the OVA peptide sequence, and purple highlights represent the Y19A mutation site.
